# Rapid Assessment of Visual Impairment in Urban Population of Delhi, India

**DOI:** 10.1371/journal.pone.0124206

**Published:** 2015-04-27

**Authors:** Noopur Gupta, Praveen Vashist, Sumit Malhotra, Suraj Singh Senjam, Vasundhara Misra, Amit Bhardwaj

**Affiliations:** Dr. Rajendra Prasad Centre for Ophthalmic Sciences, All India Institute of Medical Sciences, New Delhi, India; London School of Hygiene and Tropical Medicine, UNITED KINGDOM

## Abstract

**Purpose:**

To determine the prevalence, causes and associated demographic factors related to visual impairment amongst the urban population of New Delhi, India.

**Methods:**

A population-based, cross-sectional study was conducted in East Delhi district using cluster random sampling methodology. This Rapid Assessment of Visual Impairment (RAVI) survey involved examination of all individuals aged 40 years and above in 24 randomly selected clusters of the district. Visual acuity (VA) assessment and comprehensive ocular examination were done during the door-to-door survey. A questionnaire was used to collect personal and demographic information of the study population. Blindness and Visual Impairment was defined as presenting VA <3/60and <6/18 in the better eye, respectively. Descriptive statistics were computed along with multivariable logistic regression analysis to determine associated factors for visual impairment.

**Results:**

Of 2421 subjects enumerated, 2331 (96.3%) were available for ophthalmic examination. Among those examined, 49.3% were males. The prevalence of visual impairment (VI) in the study population, was 11.4% (95% C.I. 10.1, 12.7) and that of blindness was 1.2% (95% C.I. 0.8, 1.6). Uncorrected refractive error was the leading cause of VI accounting for 53.4% of all VI followed by cataract (33.8%). With multivariable logistic regression, the odds of having VI increased with age (OR= 24.6[95% C.I.: 14.9, 40.7]; p<0.001). Illiterate participants were more likely to have VI [OR= 1.5 (95% C.I.: 1.1,2.1)] when compared to educated participants.

**Conclusions:**

The first implementation of the RAVI methodology in a North Indian population revealed that the burden of visual impairment is considerable in this region despite availability of adequate eye care facilities. Awareness generation and simple interventions like cataract surgery and provision of spectacles will help to eliminate the major causes of blindness and visual impairment in this region.

## Introduction

Visual impairment is a global public health challenge. In 2013, a Global Action Plan was formulated by the World Health Assembly with an overall goal to reduce the prevalence of avoidable visual impairment worldwide.[1] Cataract and uncorrected refractive errors contribute to more than three-fourths of the global burden of visual impairment, both of which are avoidable.[2] In India, cataract has been reported to be the leading cause of blindness and visual impairment, as per the national blindness survey conducted in 2007.[3]

Reliable data on the prevalence of visual impairment and blindness are a pre-requisite for planning eye care services. Although population-based, detailed prevalence studies provide reliable information for goal setting, planning and starting up eye care services, they are logistically expensive, resource-intensive and time consuming. Hence, a rapid methodology may be employed, and preferred in such circumstances to estimate the burden of the problem and to provide baseline data for planning health care services using limited resources. Several rapid assessment methods related to ophthalmic epidemiology have been described earlier.[4] Rapid Assessment of Cataract Surgical Services (RACSS) is one of the earliest types of rapid assessment method employed in eye care. In RACSS, the main focus is on prevalence of cataract and availability and utilization of cataract surgical services in a particular region. Thereafter, a more comprehensive, rapid assessment method namely Rapid Assessment of Avoidable Blindness (RAAB) was developed in order to assess the prevalence and causes of visual impairment and blindness in population aged 50 years and above. The RAAB methodology focuses on blindness primarily and does not provide information on uncorrected presbyopia, spectacle use and coverage, both of which are important indicators for assessing the penetration of eye care services in a region.

To overcome these shortfalls, a novel rapid assessment methodology, known as 'Rapid Assessment of Visual Impairment (RAVI)' was described and validated in subjects aged 40 years and older.[5] This methodology has been utilized extensively in southern parts of India and an information gap exists from northern India, most notably from urban areas. In order to plan effective primary and secondary level eye care services in the Capital region of the country, the present study was designed to assess the prevalence and causes of blindness, severe visual impairment and moderate visual impairment among people aged 40 years and above in East Delhi district.

## Material & Methods

### Study Area

The district of East Delhi was selected for the RAVI survey. East Delhi is an administrative district of the National Capital Territory of Delhi in India. It is situated on the eastern banks of river Yamuna and is divided into three subdivisions namely Gandhi Nagar, Preet Vihar and Vivek Vihar. As per Census of India, 2011 the district has a population of 1,709,346 (11% of Delhi population) with a population density of 27,132 per square kilometer. The majority of the population (99.8%) in this district is urban and males comprise of 53.1% of the population. The literacy rate of the district has been reported to be 89.3%. [6,7]

### Ethics Statement

The study protocol received ethical approval from the Institute Ethics Committee, All India Institute of Medical Sciences, New Delhi, India. Clearance to conduct the survey was also obtained from the local administrative authorities. The study was explained to the local health care workers & volunteers. The examination protocol was explained to the household head and each adult participant in their local language. Written informed consent for enrollment and examination was obtained from all adults before they were included in the study in accordance with the principles of the Declaration of Helsinki. Consent was documented on the forms by the epidemiologist of the respective survey team and an impartial witness. Personal identifiers were removed from the dataset before analysis.

### Sample Size & Sampling

The sample size was calculated based on the assumed prevalence of visual impairment (presenting visual acuity of less than 6/18 in the better eye) of 15% among 40+ age group, a relative precision of 15%, 95% confidence interval, design effect of 2, power of 80% and non-response rate of 15%.

A multistage cluster random sampling technique was used for the present study. The Preet Vihar sub-district, with a total of 27 wards was randomly chosen from the three sub-districts in East Delhi district for the RAVI survey. The list of municipal wards with basic demographic data was procured from the Electoral Office, Kashmiri Gate, New Delhi. The final study area comprised of six randomly chosen wards from the selected sub-district. Each Enumeration Block (EB) as per Census 2011, India usually comprises of an approximate population of 400–500 persons. Hence, each EB comprising of a minimum of 100 adults aged 40 years and above, was taken as the sampling cluster. It was planned to cover a total of 24 of 603 enumeration blocks in the sub-district to cover an estimated sample of 2400. All the households in the randomly selected EB were covered.

### Training and inter-observer agreement

Three teams were deployed to cover 24 study clusters in East Delhi district. Each survey team comprised of an epidemiologist, an ophthalmologist, two optometrists, and two health workers. Prior to the survey, a two-day rigorous training was imparted to all team members regarding the informed consent process, standardized study procedures, cluster selection & coding, enumeration methods, clinical examination and maintenance of daily records. Inter-observer agreement among the ophthalmologists for clinical diagnosis and among optometrists for estimation of distant and near visual acuity was performed in the hospital and field setting. Good inter-observer agreement was found for all survey procedures among ophthalmologists (kappa>0.8) and among optometrists (kappa>0.85).

### Data collection and examination

The survey was conducted during January-February 2013. Mapping and finalization of cluster boundaries was done with the help of local volunteers. Compact segment sampling methodology was followed. At the start of the survey, the houses were enumerated and written informed consent was obtained from all eligible adults. The data was collected on the standard RAVI Survey Record form that focused on the avoidable causes of blindness and visual impairment in people aged 40 years and older. This form was derived from the standard RAAB form but had additional sections for near vision assessment, spectacle coverage and barriers to spectacle wear (S1 File).

The standardized RAVI Survey Record form has separate sections to record the demographic and identification details of the eligible participants. Presenting and pinhole visual acuity in each eye and the principal cause of visual impairment, if present, was also noted on the form. Distance visual acuity (VA) was measured with a logMAR‘E’ chart with five 6/18 and 6/60 optotypes on either side of the vision placard. If necessary, the distance between the subject and the chart was decreased as per standard guideline to record visual acuity worse than 6/60. If the presenting visual acuity in either eye was less than 6/18, then pinhole acuity was also noted. VA of both eyes was recorded sequentially. Detailed lens and retinal examination was done with the help of a portable slit lamp and direct ophthalmoscope, in eyes with aided pinhole visual acuity of less than 6/18. The record forms were checked for completion at the survey site itself.

### Study definitions

The definitions of visual impairment used in this study were in accordance to the categories proposed by International Classification of Diseases Update and Revision 2006 that defined visual impairment according to presenting vision.[8] The various categories of visual impairment are shown in Table 1.

**Table 1:** Categories and definition of blindness and visual impairment.

Presenting distance visual acuity in better eye		
Category	Worse than	Equal to or better than
Mild or no visual impairment	__	6/18
Moderate visual impairment	6/18	6/60
Severe visual impairment	6/60	3/60
Blindness	3/60	No light perception

In this study, uncorrected refractive error was defined as a condition when the presenting VA was less than 6/18 but improved to 6/18 or better with pinhole examination of the same eye. Cataract was defined as opacity of the crystalline lens in the pupillary area, obscuring a clear red reflex with presenting visual acuity of less than 6/18 that did not improve with pinhole. Presbyopia was defined as inability to read N8 optotype binocularly on the near vision chart. The principal cause of presenting vision less than 6/18 was recorded for each eye separately and for the individual in case of bilateral visual impairment. In cases with more than one cause for visual impairment, then as per WHO convention, the disease that was more easily preventable/treatable or correctable to achieve VA better than 6/18 was considered as the principal cause of visual impairment. For example, if the patient had cataract and refractive error, refractive error was noted as the principal disorder in the form. When there were co-existing primary disorders in the same or different eyes, the principal disorder that was most readily curable or preventable, was marked.

### Data Management & Statistical Analysis

The data was entered in specially designed Microsoft Access based database with inbuilt validation and consistency checks. Data analysis was carried out using Stata 13.0 software package (Stata Corp., College Station, Texas, USA). Qualitative data has been described as number (%) and quantitative data has been described as mean ± standard deviation as appropriate. Age and gender specific prevalence of visual impairment and their corresponding 95% confidence interval (C.I.) were calculated. Demographic association of visual impairment was assessed with age, gender and education using multiple logistic regression analysis. The results for the same were reported as adjusted Odds Ratio along with 95% confidence interval [OR (95% C.I.)]. P value of less than 0.05 was considered statistically significant.

## Results

Of the 2421 subjects enumerated, 2331 (96.3%) were examined. The response rate for examination was better among females (97.7%) than males (94.9%). Amongst the sampled population, 49.9% were females while 50.7% of the examined population comprised of females.

Amongst the people examined, nearly half of the respondents (43.8%) were aged 40–49 years, and 8.8% respondents were aged 70 years and above (Table 2). The mean age of the participants was 53.2±10.5years (males vs females: 53.9±10.3vs 52.6±10.7 years). Nearly half (48.5%) of the study population had not received any formal education.

**Table 2:** Age and Gender Distribution of People with Blindness and Visual Impairment.

	Male (n = 1149)				Female (n = 1182)				Total (n = 2331)			
Age group	Total	VI*	Blind** (WHO)	Blind (Indian)	Total	VI	Blind (WHO)	Blind (Indian)	Total	VI	Blind (WHO)	Blind (Indian)
(years)	n	n (%)	n (%)	n (%)	n	n (%)	n (%)		n	n (%)	n (%)	n(%)
40–49	468	10 (2.1)	2(0.4)	2 (0.4)	552	14 (2.5)	1(0.2)	1 (0.2)	1,020	24 (2.4)	3(0.3)	3 (0.3)
50–59	298	21 (7.0)	0 (0.0)	6 (2.0)	260	33 (12.7)	1(0.4)	2 (0.8)	558	54 (9.7)	1(0.2)	8 (1.4)
60–69	279	44 (15.8)	4(1.4)	4 (1.4)	269	64 (23.8)	7(2.6)	11 (4.1)	548	108 (19.7)	11(2.0)	15 (2.7)
≥ 70	104	33 (31.7)	3(2.9)	10 (9.6)	101	47 (46.5)	11(10.9)	15 (14.9)	205	80 (39.0)	14(6.8)	25 (12.2)
Total	1,149	108	9	22	1,182	158	20	29	2,331	266	29	51

VI = Visual Impairment

*Visual Impairment defined as Presenting visual acuity <6/18 in the better eye.

** Blindness as per WHO and Indian criteria defined as Presenting visual acuity < 3/60 and <6/60 in the better eye respectively.

### Prevalence of Visual Impairment and Blindness

Visual impairment including blindness was seen in 266 individuals with a prevalence of 11.4%(95% C.I.: 10.1,12.7). The prevalence of Moderate & Severe Visual Impairment in subjects aged 40 years was 9.2%and 0.9%respectively. The prevalence of blindness in the study population was 1.2% (95% C.I.: 0.8,1.6).

The prevalence of visual impairment was significantly higher (p = 0.03) among females (13.4%) as compared to the male participants (9.4%). Similarly, the prevalence of blindness was higher in females (1.7%) than in males (0.8%), as shown in Table 2. On applying multiple logistic regression, females were at greater odds of having visual impairment [OR = 1.4(95% C.I.: 1.0,1.9);p = 0.026]. The illiterate population was also at higher odds of having VI than the educated participants [OR = 1.5(95% C.I.: 1.1,2.1);p = 0.008].

It was observed that the prevalence of visual impairment increased with age and was noted to be maximum in people aged 70 years and above. It was 2.4% at 40–49 years of age and increased to 39.0% in participants over 70 years of age. This elderly group had nearly 25 times odds of having visual impairment (p<0.0001) than people aged 40–49 years (Table 3).

**Table 3:** Prevalence of Visual Impairment stratified by age, gender and education.

	All participants	No Visual Impairment*	Visual Impairment	Adjusted Odds ratio (95% CI)	PValue
	(n = 2331)	(n = 2065)	(n = 266)		
		n (%)	n (%)		
**Age Group (years)**					
40–49	1020	996 (97.6)	24 (2.4)	1	
50–59	558	504 (90.3)	54 (9.7)	4.4 (2.7, 7.2)	<0.001
60–69	548	440 (80.3)	108 (19.7)	9.5 (5.9, 15.1)	<0.001
≥70	205	125 (61.0)	80 (39.0)	24.6 (14.9, 40.7)	<0.001
**Gender**					
Male	1149	1041 (90.6)	108 (9.4)	1	
Female	1182	1024 (86.6)	158 (13.4)	1.4 (1.0. 1.9)	0.026
Education Level					
Any education	1201	1119 (93.2)	82 (6.8)	1	
No formal education	1130	946 (83.7)	184 (16.3)	1.5 (1.1, 2.1)	0.008

CI = Confidence Interval

*Visual Impairment defined as Presenting visual acuity <6/18 in better eye

The prevalence of visual impairment and blindness in the older age group (≥50 years) was 18.5% (95% C.I.: 16.4, 20.6) and2.0% (95% C.I.: 1.2,2.7). When the Indian definition of blindness (presenting visual acuity less than 6/60 in better eye)was used, the prevalence of blindness in the 40+ and 50+ population was reported to be 2.2% (95%C.I.:1.6,2.8) and 3.7% (95% C.I.: 2.6,4.7) respectively.

### Causes of Visual Impairment& Blindness

**Fig 1:**
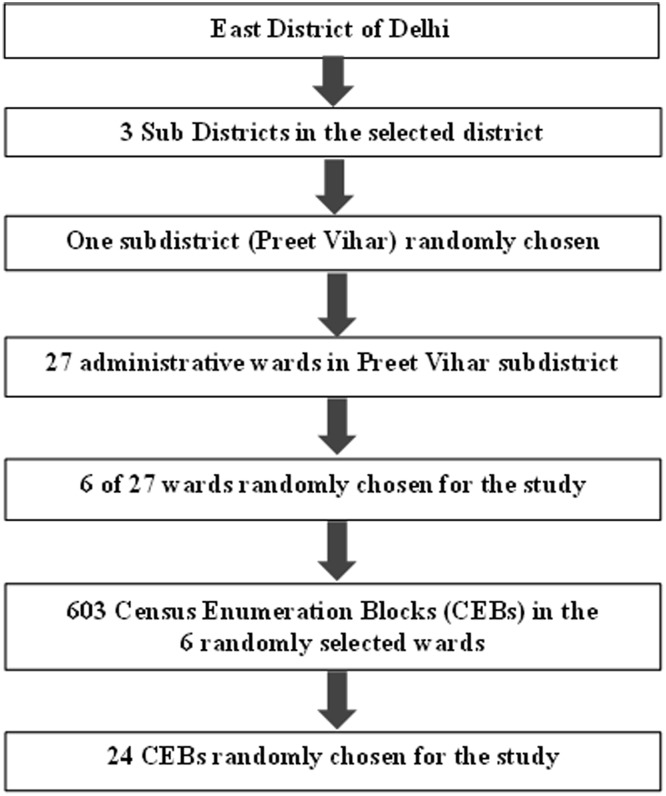
Steps of Multi-stage sampling employed for the study.

Uncorrected refractive error accounted for nearly half of the cases (53.4%) with visual impairment. Nearly one-third (33.8%) of the people with VI had cataract (Table 4). Posterior segment disorders including diabetic retinopathy, corneal diseases, cataract surgical complications and uncorrected aphakia together were responsible for nearly 10% of VI in this region. When causes of severe visual impairment were analyzed, it was observed that cataract was responsible for 77.3% and uncorrected refractive errors were responsible for 13.6% of severe visual impairment (Fig 1).

**Table 4:** Principal Causes of Visual Impairment, Severe Visual Impairment and Blindness in the Study Population.

Principal Cause	VI^#^	SVI	Blind (WHO)**	Blind (Indian)**
	n(%)	n(%)	n(%)	n(%)
**Uncorrected Refractive Error**	142(53.4)	2 (9.1)	3(10.3)	5 (9.8)
**Cataract, untreated**	90(33.8)	18 (81.8)	19(65.5)	37 (92.5)
**Surgical Complication**	9(3.4)	1 (4.6)	1(3.4)	2 (3.9)
Posterior Segment Diseases*	8(3.0)	1 (4.6)	3(10.3)	4 (7.8)
**Diabetic Retinopathy**	5(1.9)	0(0.0)	0(0.0)	0 (0.0)
**Corneal Opacity**	4(1.5)	0(0.0)	2(6.9)	2 (3.9)
**Aphakia, uncorrected**	3(1.1)	0(0.0)	1(3.4)	1(1.9)
**Glaucoma**	2(0.8)	0(0.0)	0(0.0)	0(0.0)
**ARMD**	2(0.8)	0(0.0)	0(0.0)	0(0.0)
**Phthisis bulbi**	1(0.4)	0(0.0)	0(0.0)	0(0.0)
**Total**	**266(100)**	**22 (100.0)**	**29(100)**	**51 (100.0)**

*Excluding Diabetic Retinopathy and ARMD;

VI = Visual Impairment; SVI = Severe Visual Impairment; WHO = World Health Organization; ARMD = Age Related Macular Degeneration

^#^Visual Impairment defined as Presenting visual acuity <6/18 in the better eye.

** Blindness as per WHO and Indian criteria defined as Presenting visual acuity < 3/60 and <6/60 in the better eye respectively.

Cataract was the single largest cause of bilateral blindness in this region (Table 4). Amongst the total blind population, 65.5% were blind due to cataract and 10.3% due to uncorrected refractive error. Trachomatous keratopathy and corneal scars together were responsible for 6.9% and posterior segment diseases were responsible for 10.3% of all bilateral blindness. The causes for blind eyes (225 eyes) were observed to be distinct and cataract (55.6%) posterior segment diseases (22.7%) and corneal scars (7.6%) were responsible for majority of the visual impairment in these blind eyes.

### Prevalence of Refractive error, presbyopia and spectacle coverage

Uncorrected refractive error was present in 275(11.8%; 95% C.I.:10.5,13.1) people aged 40 years and older. Out of a total of 2331 individuals who were examined, 468(20.1%) were using spectacles for distance. Out of these, 314 (67.1%) were using bifocal correction while the rest were using spectacles for distance vision only (S1 File).

Nearly one-third of the study population (798;34.2%;95% C.I.:32.3,36.2) had presbyopia, of which 60.5% (483) were females. The prevalence of presbyopia was higher among females (40.9%; 95% C.I.:38.1,43.7) as compared to males (27.4%;95% C.I.: 24.8,29.9). Presbyopic spectacles were being used by 397 of 798 (34.1%) presbyopes in the study population.

## Discussion

The present study reporting the magnitude of visual impairment and blindness in the adult population of New Delhi was conducted as part of Vision Delhi-Comprehensive Community Eye Care Initiative. Since typical epidemiological, population-based studies are time-consuming, resource-intensive and difficult to implement, an innovative, rapid assessment methodology (RAVI) was utilized during this survey.[5] To the best of our knowledge, this is the first study highlighting the burden of visual impairment and blindness in the Indian capital.

The prevalence of visual impairment in people aged 40 years and older was 11.4% (95% C.I.: 10.1, 12.7) in our study. This is comparable to previously conducted population-based studies in China and southern India (Table 5).[9,10] In India, there are wide regional variations in the reported burden of visual impairment.[3,10–13] The prevalence of visual impairment across the country ranges from 14.3% to 42.1% (Table 5). An important point to note here is that most of these studies were conducted in the 50+ population, where the prevalence of visual impairment, including blindness is higher. Studies conducted in countries other than India also demonstrated a considerable variation in prevalence of VI.[9,14,15] This could be attributed to differences in study design, case definitions, study methodology, sample size and age group included.

**Table 5:** Magnitude of Visual Impairment reported in population-based studies published in the past decade (2004–2014).

Authors & Year of publication	Place of study	Age Group	Sample size	Prevalence of VI**(%)
		(yrs)	(examined)	(95% Confidence Interval)
**Studies from India**				
Patel S et al, 201411	Sindhudurg district, Maharashtra	≥50	2747	33.6 (30.5,36.7)
MarmamulaSet al, 201310	3 districts of Andhra Pradesh	≥40	7378	14.3 (13.5,15.0)
Murthy GV et al, 201012	Navsari district, Gujarat	≥50	4738	18.0*
Neena J et al, 20083	16 districts of India	≥50	40447	24.8*
Vijaya L et al, 2006`13	2 districts of Tamil Nadu	≥40	3924	42.1*
Present Study	East Delhi district	≥40	2331	11.4(10.1,12.7)
Present Study	East Delhi district	≥50	1311	18.5 (16.4, 20.6)
**Global Studies**				
Robinson B et al, 201314	Brandfort city, Canada	≥40	768	2.7 (1.8,4.0)
Zhu M et al,20139	Baoshandistrict, China	≥60	4545	8.8(8.0,9.7)
Ramke J et al, 200715	2 districts of Timor Leste	≥40	1414	23.2*

VI = Visual Impairment

*Confidence interval not available

**Visual Impairment defined as Presenting visual acuity <6/18 in better eye

The prevalence of blindness in our study population was 1.2% (95% C.I.: 0.8, 1.6). As compared to the National RAAB (Rapid Assessment of Avoidable Blindness) survey of India conducted in 2007,[3] the prevalence of blindness is low in the capital and this is heartening to report. The National RAAB survey conducted amongst the 50+ Indian population reported the prevalence of blindness(presenting visual acuity less than 3/60 in the better eye) as 3.6%.[3] In the present study, the reported prevalence of blindness in the 50+ population was 2.0% (95% C.I.: 1.2, 2.7). The reasons for lower prevalence of visual impairment and blindness in the national capital could be attributed to successful implementation of health care programmes, availability and accessibility of health care services and improved affordability of the population. Even then, efforts are still needed in this region to completely eliminate the burden of avoidable blindness. To achieve elimination of avoidable blindness by 2020, diseases like cataract, refractive error, uncorrected aphakia and corneal opacities need to be managed timely and effectively.

Indian studies on blindness frequently use the Indian definition of blindness (presenting visual acuity of less than 6/60 in the better eye). For the purpose of comparison, the prevalence of blindness in the 50+ population as per the Indian definition was 3.7% in the present study. In the last decade, various RAAB and RAVI studies have been conducted in the Indian subcontinent. Using the above definition of blindness and the same subset of population, RAVI survey done in the state of Andhra Pradesh during 2011–12 reported 9% prevalence of blindness while RAAB conducted in three tribal regions of this state during 2014 revealed a prevalence of 5.2%.[10,16] RAAB conducted in Sindhudurg district of Maharashtra demonstrated prevalence of blindness as 14.5% and prevalence of 7.4% in Kolar district of Karnataka.[11,17]

The prevalence of visual impairment in our study population was affected by age and older age was reported to be a major risk factor for the same. The elderly age group (≥ 70 years) had nearly 25 times more risk of having visual impairment when compared to adults in the fifth decade. The same trend was also exemplified in other population-based studies from India and adjoining developing countries.[3,18–21] Illiteracy, independently, was a significant risk factor for blindness and visual impairment in this urban population. These results are consistent with other studies conducted in the South East Asia region.[18–21] This could be explained by poor socio-economic status, poor access to health care services and also due to barriers related to wearing spectacles in this segment of the society. Hence, community programmes should aim at enhancing health literacy related to eye diseases, especially targeting illiterate segments of the population.

The prevalence of visual impairment was significantly higher among females as compared to males (p = 0.026) in the study population. Similar findings have been reported by other population-based studies.[3,10] The reasons for this could be the disadvantaged position of females in our society which results in poor accessibility to eye care services.

Cataract and refractive error are still the main ocular diseases that contribute to visual impairment (87.2%) and blindness (75.8%) in this North Indian population. Similar to other studies done in India and other neighbouring countries with a developing economy, cataract and uncorrected refractive error continue to be the main causes of visual impairment and blindness.[18–23] Despite the younger age group included in our study, the pattern of visual impairment and blindness is similar to previously conducted blindness surveys. Hence, measures to increase cataract surgical services and provision of good quality spectacles are needed in this region as well. Corneal opacities contributed significantly (6.9%) to the burden of blindness in the study population. A higher proportion of blindness (10.3%) was caused by posterior segment disorders, consistent with some recent studies conducted in India.[16,17]

The main strength of this study remains the high response rate and coverage of the enumerated survey population. Over 95% of the randomly sampled population participated in the study indicating that the results are representative of the standard urban population of Delhi. We would like to highlight that the sample size was not adequate for ascertaining the true prevalence of blindness in this population. However, the sample size was appropriate for assessing the primary objective of the study which was to determine the population prevalence of visual impairment. It is possible that we overestimated uncorrected refractive error as a cause of visual impairment as all cases were labeled as refractive error when visual acuity improved with pin hole, in spite of presence of lenticular or corneal opacity. Dilated retinal evaluation and slit lamp biomicroscopy was performed by experienced ophthalmologists in the field setting, and this imparts additional value to the study results. Very few studies conducted in the population setting are carried out so comprehensively and in an exhaustive manner. Results of other RAVI studies available from South India were based on torch light examination done by ophthalmic technicians.[5,16,23]

This is the first study to exemplify the burden of visual impairment in the urban population of the national capital. Although the current study was conducted in the East district of Delhi, the results can be extrapolated to the rest of Delhi. The study findings can be utilized for evidence-based planning and regular monitoring of ongoing cataract intervention programmes and refractive error services in the national capital. This is one of the mandates of Global Eye Health Action Plan 2014–2019.[1] The World Health Assembly recommended that methodologically sound and representative surveys on the prevalence of visual impairment, including blindness should be conducted on periodic intervals in all member countries. Good quality eye care services along with health education and awareness programmes need to be undertaken to improve the health seeking behavior and increase uptake of these services in this region of the country.

## Supporting Information

S1 File Rapid Assessment of Visual Impairment, Delhi: A Report. (PDF)
