# Supplementary material for: Rapid Assessment of Visual Impairment in Urban Population of Delhi, India
Source: PLoS One. 2015 Apr 27;10(4):e0124206. doi: 10.1371/journal.pone.0124206 (PMC4411096; doi:10.1371/journal.pone.0124206)
Supplement: S1 File — (PDF) [file pone.0124206.s001.pdf]

Rapid Assessment of Visual Impairment- Delhi

Report

2013

Department of Community Ophthalmology, Dr. R. P. Centre
AIIMS, New Delhi
Conducted in Partnership with Sightsavers

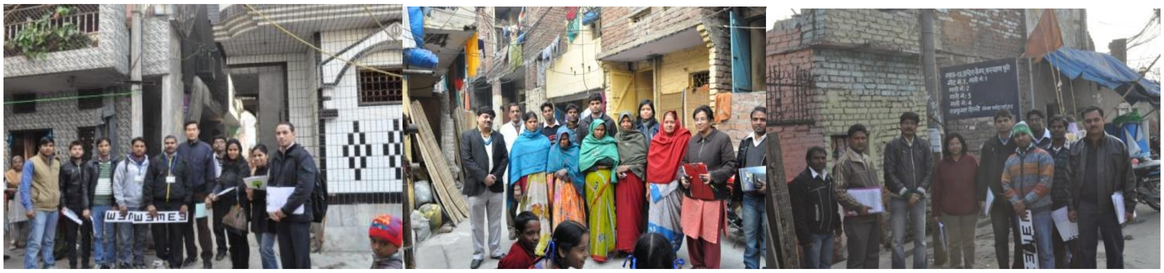

Rapid Assessment of Visual Impairment, Delhi 2013

Report

Editors

Praveen Vashist
Sumit Malhotra
Suraj S. Senjam
Noopur Gupta

1. BACKGROUND

Visual impairment is a global public health challenge. Cataract and uncorrected refractive errors contribute to more than three-fourths of the burden of visual impairment globally and are also the leading causes of blindness worldwide. In India, uncorrected refractive error is the leading cause of visual impairment and cataract is the leading cause of blindness. Uncorrected presbyopia affects nearly 410 million people globally. Most of the cases of refractive errors and presbyopia can be easily corrected with spectacles, and cataract can be addressed by surgery.

Reliable data are a pre-requisite for planning eye care services. Although population-based, detailed prevalence studies provide reliable information for goal setting, planning and starting up eye care services, they are logistically expensive, resource intensive, time consuming and require expertise in epidemiology. Hence, a rapid methodology is required to estimate the burden of the problem and also to provide baseline data to plan the services using limited resources. Several rapid assessment methods have been described earlier. Rapid Assessment of Cataract Surgical Services (RACSS) is one of the earliest of rapid assessment methods used in eye care. In RACSS the main focus is prevalence of cataract and cataract surgical services. The Rapid Assessment of Avoidable Blindness (RAAB) is a rapid assessment method that is more comprehensive and includes all the causes of visual impairment in 50+ population with a focus on cataract. It does not provide information on spectacle use and coverage, both of which are important indicators for assessing the penetration of eye care services in the region. The RAAB also does not provide information on uncorrected presbyopia, which contributes to a significant proportion of near visual impairment globally.

A novel rapid assessment methodology, titled 'Rapid Assessment of Visual Impairment (RAVI)' was used to investigate the prevalence and causes of visual impairment and presbyopia in subjects aged 40 years and older. This report describes the detailed methodology and study procedures of Rapid Assessment of Visual Impairment (RAVI) survey under the Vision Delhi project supported

by Sight Savers. This report can be used for the planning and management of eye care services in East Delhi district.

2. METHODOLOGY

2.1 OBJECTIVES

1. To assess the prevalence of blindness, severe visual impairment and moderate visual impairment among people aged 40 years and above in East Delhi district.
2. To determine the main causes of blindness and visual impairment.
3. To assess the spectacle coverage for refractive errors & presbyopia and determine the barriers and facilitating factors responsible for uptake of spectacles.
4. To determine the met and unmet need for presbyopia in this urban population aged 40 years and above.
5. To determine the prevalence of aphakia and/or pseudophakia and cataract surgical coverage in this area.
6. To assess the visual outcomes after cataract surgery.
7. To assess barriers responsible for not availing cataract surgical services.
8. To assess indicators for cataract surgical services (age at time of surgery, place, cost and type of surgery, cause of visual impairment and level of satisfaction after cataract surgery) in the study area.

2.2 EXPECTED OUTCOME

All indicators are subdivided by sex and by age group. The indicators thus obtained can be used as baseline information for the formulation of eye care programmes and for regular monitoring of ongoing cataract intervention programmes and refractive error services in this region.

2.3 SAMPLING

2.3.1 SURVEY POPULATION

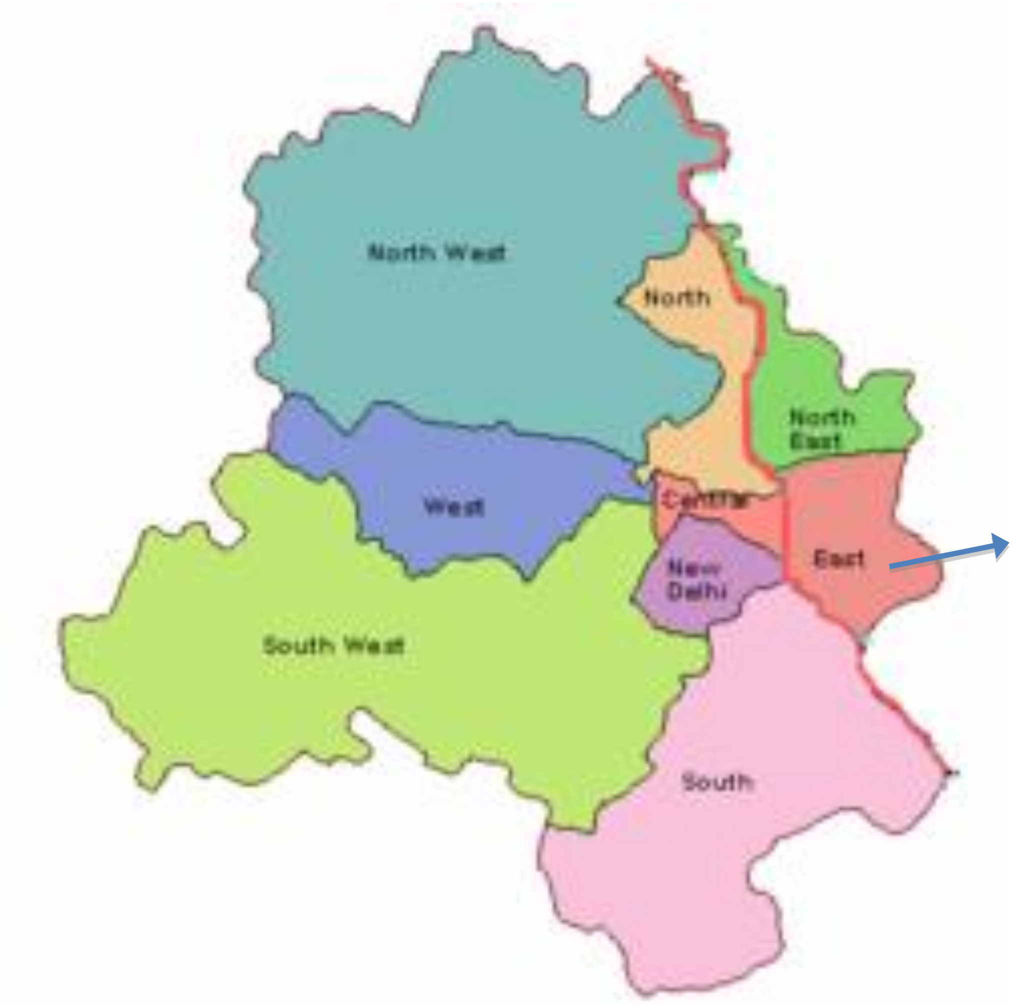

The district of East Delhi was selected for the RAVI survey. The East Delhi district is situated on the eastern banks of river Yamuna that divides Delhi in two parts. As per Census of India, 2011 the district has a population of 1,707,725 (11% of Delhi population) with 59 municipality wards and a population density of 27,132 per square kilometer. The majority of the population (99.8%) in this district is urban. The sex ratio of the district is 883 with a decadal growth rate of 16.68%. The literacy rate of the district is 88.75%.

2.3.2 SAMPLE SIZE

The sample size was calculated based on an assumed prevalence of visual impairment (presenting visual acuity of less than 6/18 in the better eye) of 15% among 40+ age group, a

relative precision of 15%, 95% confidence interval, design effect of 1.5, power of 80% and non-response rate of 15%. The final calculated sample size was **2300** persons.

2.3.3 SAMPLING METHODOLOGY

In urban regions of India, the district is divided into municipal wards. A list of all the municipal wards in East Delhi district was procured from the Electoral Office, Kashmiri Gate. In the first stage of sampling, three wards were randomly chosen in the East Delhi district. These wards were Kalyanpuri, Khichripur and Trilokpuri. In these three wards, 57 enumeration blocks were listed. Each Enumeration Block (EB) /sub-block as per Census 2011 usually comprises of 80-100 households with an approximate population of 500-700 persons. As the approximate population of individuals aged 40 years and above is around 25%, an enumeration block would then, comprise of nearly 100-125 eligible adults aged 40 years and above and hence each EB was taken as the sampling cluster. It was planned to cover a total of 24 such enumeration blocks to cover the estimated sample of 2400. A total of eight enumeration blocks were randomly selected from each of the three wards (Table 1).

In the second stage of sampling, the first household was randomly selected as generated through computer random tables. The next household whose door was closest to the previous household already covered was selected subsequently. All the households in the randomly selected EB were covered. In each cluster, 100 individuals aged 40 years and above were recruited for examination.

Table 2.1: Randomly selected clusters of East Delhi district where RAVI was conducted

Var 1	Var 2	Var 3	**Cluster code**	Var 6
219	DMC (U) 7001	East 04	**01**	6/86-89,101-120,187-195,257-280 Block No. 6, Khichri pur colony
214	DMC(U) 7001	East 04	**02**	H.No. 161 – 179 221-250 Block No 3Khichripur
219	DMC (U) 7001	East 04	**03**	CN 27-98, CN 1-16, T. Huts Block 7, Khichripur
214	DMC(U) 7001	East 04	**04**	H.No. 251-280191-220Block No. 9 Khichripur
213	DMC(U) 7001	East 04	**05**	Khichari Pur, Near Block No-1, C.No-1 to C-85, 1/2 To 1/30
219	DMC (U) 7001	East 04	**06**	8/181-220, 251-280, Block No. 8 Khichri Pur Colony
		East 04	**07**	Block 7, Khichripur
		East 04	**08**	Block 8, Khichripur
213	DMC(U) 7001	East 04	**09**	Kalyan Puri, Block -18, Indra Camp, C.No-701 to 840
213	DMC(U) 7001	East 04	**10**	Kalyan Puri, B.No-13, H.no- 35 to 149
213	DMC(U) 7001	East 04	**11**	Kalyan Puri , Block no-18, H.no 228 to 246, 262 to 280, 286 to 318, 334 to 356.
213	DMC(U) 7001	East 04	**12**	Kalyan puri, B.No-13, H.No- 61 to 100.
213	DMC(U) 7001	East 04	**13**	Kalyan Puri, Blk-17, J.J.Colony, Indra Camp, C.no-988-1100
213	DMC(U) 7001	East 04	**14**	Kalyan Puri, Blk No-15, H.no- 01 to 15, 16 to 30,101 to 110,111 to 120.
		East 04	**15**	Kalyan Puri, Blk- 15
		East 04	**16**	Kalyan Puri, Blk No-18
209	DMC(U) 7001	East 04	**17**	TRILOK PURI BLCK -13 H.NO.111-180
210	Dallupura C.T.8057	East 04	**18**	Trilokpuri, Trilokpuri Block-55/61- 5/75, 5/96- 5/110
210	Dallupura C.T.8057	East 04	**19**	Trilokpuri, Trilokpuri Block-6 6/461- 6/500
210	Dallupura C.T.8057	East 04	**20**	Trilokpuri, Trilokpuri Block-11 11/101- 11/130 ,11/191 - 11/210
211	Chilla Saroda Banger C.T. 8058	East 04	**21**	Block No-35 Extra Trilokpuri H.No 1-28
210	Dallupura C.T.8057	East 04	**22**	Trilokpuri, Trilokpuri Block-1515/71- 15/140
		East 04	**23**	Block 13, Trilokpuri
		East 04	**24**	Block 22, Trilokpuri

2.4 STUDY TOOLS AND SURVEY PROCEDURES

The RAVI Survey Record Form contains eight different sections which are as follows:

A. General Information & Consent form

B. Vision – presenting vision and pinhole vision

C. Lens Examination

D. Principal cause of presenting vision < 6/18 in any eye

E. Details about cataract operation if applicable

F. Why cataract operation was not done

G. Spectacle Use

H. Barriers to use of spectacles

The RAVI Survey Record Form focuses on the avoidable causes of blindness and visual impairment in people aged 40 years and older. Cataract and refractive error are major treatable causes of visual impairment and gets much emphasis.

Posterior segment eye disease (e.g. glaucoma, ARMD and diabetic retinopathy) is usually more difficult to diagnose with the limited diagnostic facilities used in this rapid assessment.

The RAVI Survey Record has been designed for use by ophthalmologists, residents in ophthalmology and experienced paramedical ophthalmic staff. The examinations for all sections, except Section D (presenting cause of visual acuity <6/18), can be completed by auxiliary personnel, such as nurses or ophthalmic assistants, adequately trained for this purpose. Examinations for section D must be completed by the ophthalmologist or ophthalmic clinical officer. It is important that the examinations are conducted following the same procedures and by using the same equipment for all persons. When experienced staff and portable slit lamps are available, a detailed lens examination with portable slit lamp and mydriasis is recommended for all eyes with a presenting VA less than 6/18, not improving with pinhole.

Instructions for completing forms

Boxes need to be filled with a number, circles have to be tick marked or made black and on lines, a text has to be written. Always use a **black pen** to fill the records and write clearly. It is important that the form is clearly marked so that the data entry person does not get confused.

If an error is made, use a **red pen** to make a single line across the correction. The correction should be noted in red and signed and dated by the concerned person.

Section A: General Information

The selection of 24 clusters is given in Table 1. For each eligible person, a RAVI Survey Record Form has to be completed, whether the person is examined, is absent, refused examination or was unable to communicate.

Item Instructions

Year: Enter year of examination.

Month: Enter month of examination.

Survey area & code: A defined geographical or administrative area, such as a ward/enumeration block with pre-designated codes will be entered. Write the name and a two-digit ID code number (01-18).

Individual no.: Write the sequential number of the person examined in the selected cluster as they are included during the study. This may vary from 001 to 150 and does not denote the prson no. in the household but in the total cluster. A total of 100 eligible individuals need to be examined and completed survey record form of 100 people per cluster is essential.

House no.: The household number as given by the supervisor is noted. This number can range from 01-99 and is study specific and does not relate to the postal address of the household.

Name: Person name, to be written in English language, as appropriate. This item will not be included in the data processing, but may be useful to trace people for follow up (if needed).

Sex: Mark the appropriate circle: male (1) or female (2).

Age: Record age in years; estimated, if no official certificate available. For ages of 40 to 98, use the age in years; for ages of 99 or higher, write 99. The RAVI software will not accept any age below 40.

Educational status: This field generates data on the educational status of the 40+individual.First ask whether the respondent went to school. If he/she says “no”, ask “can you read and write”. If answer is “No” record “illiterate”, code 00; if answer “yes” record “can read and write” code 50. For all those who attended school, ask about number of years of schooling and also whether extra education was received after leaving school. Code number of years of schooling if NO extra education after school. If extra education after school code the final level attained e.g. diploma=14, graduation=15, post-graduation=17, professional e.g. doctor, engineer, lawyer=18. Example: person reported 8th grade only, record 8; person reported 8th grade plus two years diploma, record 14. Person reports 12 years

schooling and MBBS graduation training for becoming dostor, record 18. '99' is recorded when the educational status is not known.

Optional: This field may be left blank.

Examination Status: Mark

• 'Examined' (1) when a subject can be examined.

• 'Absent' (2) if a resident is not present during the survey period, even after repeated visits.

• 'Refused' (3) when a resident refuses to be examined.

• 'Unable to communicate' (4) when a resident is profoundly deaf, has dementia or psychiatric illness so that it is not possible to test their visual acuity.

For all such individuals who cannot be examined (Option 2, 3, 4)-Section E needs to be filled (detailed later)

Section B: Vision

In section B, fill in the presenting and pinhole visual acuity for each eye separately.

Equipment needed: simplified 'E' chart, pinhole occluder and rope to measure distance of 6 metres.

Method: VA is tested using the simplified illiterate `E' chart with available correction. Visual acuity is measured with a chart with "E" optotype of size 18 of the Snellen chart on one side and an "E" optotype of size 60 on the other side at 6 or 3 metres distance with available correction. This is best done in full daylight, in the courtyard or on the street. Distance is measured with a black ribbon of 6-metre length, with a ring/knot at both ends and one in the middle (3 meters). The examiner puts one end around a finger and keeps that hand against the chest; the examinee does the same with the end with clip at the other end of the tape. First the right eye is examined, while the left eye is covered with the palm of a hand or an occluder, either by the examinee, or by a helper. The examinee should stand in the shade or with his or her back to the sun, while the E chart is kept up in clear daylight. Vision is tested separately for each eye. If a patient usually wears distance glasses, these should be worn during visual acuity measurement.

THIS SHOULD ALSO BE RECORDED IN THE FORM. NO FIELD IS TO BE LEFT BLANK.

First the 'E' chart is shown from nearby, the procedure is explained and the examinee is instructed to point in the direction of the open ends of the "E". Then the "E" optotype of size 6/60 is shown first at a distance of 6 metres. It is advisable to start with the larger E to test if the patient understands the procedure. If they can see the E size 60 at 6 metres (6/60), change to the E size 18 at 6 metres distance (6/18). If they cannot see the E size 60 at 6 metres, change to size 60 at 3

metres (3/60). If the "E" of size 60 cannot be seen at 1 metre distance, check with a torch in semi-dark condition (inside the house) whether the person has perception of light (PL+) or not (PL-). Each chart has five optotypes of equal sizes. The criteria for vision at certain level are 4 correct consecutive showings, or 4 correct out of 5 showings. An eye with a **presenting VA better than 6/18 does not need to be examined with pinhole** – just mark code 1 for pinhole vision. Any eye with a presenting VA less than 6/18 has to be examined for acuity with a pinhole as well. Mark the VA obtained with the pinhole. If the person wears spectacles, place the pinhole in front of the spectacles. In some cases, the available correction is not the optimal correction. Vision with pinhole correction cannot be worse than presenting vision.

Table 1: Categories and definition of blindness and visual impairment

**Presenting distance visual acuity**		
**Category**	**Worse than:**	**Equal to or better than:**
Mild or no visual impairment		6/18
Moderate visual impairment	6/18	6/60
Severe visual impairment	6/60	3/60
Blindness	3/60	No light perception

Item Instructions

Glasses Mark the appropriate circle for distance glasses only. If the person wears glasses for distant vision these should also be used during the vision testing. Please check for plano glasses, specially if patient is wearing bifocal with no distance correction. Please record no distance glasses being used in such cases.

Presenting vision in right and left eye

Mark the appropriate circle for each eye. Only one entry is allowed.

Pinhole vision in right and left eye

If presenting vision is 6/18 or better, then pinhole vision is the same. All eyes with VA<6/18 should be also tested with pinhole. If vision was tested with glasses, these should be used here as well. **Place the pinhole in front of the patient's glasses. Don't remove the glasses for recording pinhole vision**

Section B1: Near vision

Near vision is to be recorded in all subjects with both eyes open. Binocular near visual acuity is measured in each individual using LogMAR near vision E-chart kept at a distance of 40 cm. Both

unaided and aided near vision was assessed, if the subject was using spectacles. Near vision is recorded whether the person can read N8 unaided, and similarly if spectacles were available.

Section C: Lens Examination

a) Standard lens examination

In Section C, only one circle must be marked for each eye. If the lens in both eyes is normal, the circle left of code (1) of each eye must be marked. Examine the lens in each eye and mark your observations in Section C:

Normal lens or minimal lens opacity; unlikely to cause reduction of visual acuity.

Clear or minimal dark shading of the red reflex.

Obvious lens opacity present,

Lens absent (aphakia),

IOL implanted without posterior capsule opacification or IOL implanted and posterior capsule opacification present.

If you cannot see the lens because of corneal scarring, Phthisis bulbi or other causes, mark "No view of lens".

In cases if lens opacity is present, but pinhole VA is better than 6/18, then the principal cause of vision loss is refractive error and not lens opacity.

When the examined eye does not improve to 6/18 or better with pinhole examination, the pupil is dilated with a short-acting mydriatic (tropicamide 0.5%) eye drop. Two drops five minutes apart should be applied. In the following conditions, the pupil should not be dilated:

- Very shallow anterior chamber, where an angle-closure glaucoma attack could be precipitated.
- Presence of obvious white cataract where the fundus would not be visible even after dilatation.
- Presence of large corneal opacity, or occlusio pupillae.

Once dilated, the lens (intraocular lens if present), the posterior capsule and the anterior vitreous are examined with the slit lamp in a semi-dark room.

Section D: Main and principal cause of presenting vision less than 6/18

This section is completed for all eyes. The abnormality causing low vision or blindness should be marked. Examination with illuminated loupe as well as direct ophthalmoscope is recommended; this should be consistently used or consistently not used throughout the survey. This also applies when a handheld slit lamp and mydriasis is used.

The completion of this section can be divided into two activities: (1) for each eye, assess and

mark one principal disorder that is responsible for visual loss in that eye; (2) mark one principal disorder responsible for or contributing to visual loss in the person. If the VA was 6/18 or better in the eye then mark 'not examined – can see 6/18' (code 14).

Mark the principal disorder responsible for visual loss in each eye as well as in the individual **(better eye)** after considering disorders in either eye, which are **most amenable to treatment or prevention**. When there are two disorders, one of which is secondary to the other, the **primary is to be selected as the principal disorder**. For example, if the patient has cataract secondary to glaucoma, glaucoma is the principal disorder. When there are co-existing primary disorders in the same or different eyes, mark as the principal disorder that which is most readily curable or, if not curable, that which is most easily preventable. The following is a recommended ranking of the disorders with respect to these criteria:

1. Refractive error
2. Uncorrected aphakia
3. Cataract
4. Surgery related complications
5. Preventable corneal opacities and phthisis
6. (Primary) glaucoma
7. Other posterior segment disorders.

The ranking may be modified to suit particular local circumstances. Once the disorders and underlying causes have been marked for each eye, an assessment is made of the principal cause of low vision in the person.

Item Instructions

Refractive error: Phakic eyes with VA< 6/18, improving with pinhole or optical correction to 6/18 or better.

Cataract: untreated obvious lens opacity, obscuring a clear red reflex, which is likely to affect vision. Do not mark this option in cases of minor opacities, unlikely to affect vision.

Aphakia: uncorrected Aphakia (absence of lens from the central pupil), improving with correction or pinhole to 6/60 or better. For aphakia where VA does not improve with proper correction, other causes of visual loss should be determined and recorded appropriately, while uncorrected aphakia should not be marked.

Surgical complications: If there is evidence that a surgical procedure has led to a blinding condition, e.g., secondary glaucoma, then this box should be marked.

Uncorrected aphakia must be recorded as above.

Trachoma: marked in cases with central corneal scarring in the presence of at least one of the following signs of trachoma: trichiasis / entropion;

Phthisis: Small shrunken globe due to trauma or severe infection.

Other corneal scar: Leucoma, staphyloma, or other easily visible corneal opacity present over the pupil without any signs of trachoma.

Globe abnormality: Microphthalmos, anophthalmos, enucleated eye.

Glaucoma: Mark if any of the following suggested criteria apply:

- Known case of diagnosed glaucoma and vision loss is due to glaucoma
- the eye is stone hard on digital palpation;
- an afferent pupil defect and corneal oedema;
- the vertical cup-disk ratio is 0.8 or greater.

This is not a complete diagnosis for glaucoma, but only used for the purpose of this survey, since tonometry and testing of visual fields is not practical under field conditions and glaucoma is not the focus of this survey.

Diabetic retinopathy: This diagnosis applies only for persons with confirmed diabetes. The retina shows either:

- proliferative retinopathy (growth of new blood vessels with or without haemorrhages), or;
- diabetic macular oedema (extensive swelling of the central retina).

Age-Related Macular Degeneration (ARMD):

ARMD refers to obvious or severe pigment disturbances at the macula from what is considered “normal” in the absence of other known causes. Check if any of the following suggested criteria apply:

- the pigment epithelium is disturbed by atrophy, or proliferation(mottling);
- presence of drusen (yellow colloid-like dots);
- swelling or oedema of the central retina;
- circinate exudates;
- Haemorrhage;
- Macular hole.

Other posterior segment or CNS disorder:

If the VA<6/18 cannot be attributed to any of the above mentioned causes, but a specific cause can be identified then use this diagnosis.

Not examined: Mark if the patient has vision of 6/18 or better in this eye and there was no indication to examine.

Once the disorders and underlying causes have been marked for each eye, an assessment is made of the principal cause of low vision in the person.

Section E: Details about cataract operation

This section is only filled in for people who have undergone cataract surgery.

Ask operated patients about their age at the time of cataract surgery. Ask them where the operation was conducted: in a government, charitable or private hospital, in an ‘eye camp’ (surgery performed by qualified ophthalmic staff in an improvised operation theatre) or in a ‘traditional setting’

Mark ‘Non IOL’ if the patient did not get an IOL implanted at the time of surgery. Mark ‘IOL implant’ for PC-IOL and for AC-IOL, also when these IOL’s are dislocated. Mark ‘Couching’ if there is evidence of dislocation of the lens and iris tremulousness, or if couching is ascertained during interview. Ask operated patients whether they paid anything for the cost of surgery, whether the operation was free, partially free or paid. Costs on transportation, food or accommodation should not be counted.

If the VA is less than 6/18 after cataract surgery, try to assess the cause of this result. If the patient did not regain full sight after an uncomplicated surgery because another eye disorder in the same eye caused loss of vision as well, then mark ‘Ocular comorbidity (Selection)’. If the borderline or poor outcome is due to complications during cataract surgery, mark ‘Operative complications’. If the vision after cataract surgery can be improved with pinhole, then mark ‘Refractive error’. Uncorrected aphakia should also be marked as refractive error for this question. Finally, in case of initial good outcome and subsequent vision loss due to postoperative capsule opacification or retinal detachment, mark ‘Long term complications’.

If the VA is 6/18 or better, or if the loss of vision after surgery is caused by another condition than cataract surgery, mark ‘Not applicable, can see 6/18’. Finally, ask patients whether they are satisfied with the results of cataract.

Section F: Why cataract operation has not been done

Section F of the RAVI Survey Record shows a list of the most common barriers to cataract surgery. This section is only filled in for people who have an obvious lens opacity and visual impairment or blindness (VA<6/18 in one or both eyes with pinhole).

Not all patients who are blind due to cataract will present themselves for operation. Many patients are not operated for a variety of reasons. These can be poor accessibility, costs, fear of operation, etc. Knowing these barriers makes it possible to address them effectively and thereby increase the utilization of cataract surgical services.

Ask people with obvious lens opacity and visual impairment or blindness (VA<6/18 in one or both eyes with pinhole) the standard question: “Why have you not been operated for cataract?” Match the answer of the patient with the barriers mentioned in the list and the answer closest to the patient’s answer should be marked. Mark at least one and a maximum of two barriers.

1 Unaware that treatment is possible
2 Believes it to be destiny / God's Will
3 Told to wait for cataract to mature
4 Surgical services not available or very far
5 Don't know how to get surgery
6 Cannot afford operation
7 No one to accompany
8 No time available / other priorities
9 Old age and need not felt
10 One eye adequate vision / need not felt
11 Fear of operation
12 Fear of loosing eye sight
13 Other disease contra-indicating operation

G. **Details about Spectacle Use-** In participants who used spectacles for distance or for near vision, this section was filled by the optometrists. The participants were asked about the duration of spectacle wear and related practices. The cost and source of the spectacles was enquired and the level of satisfaction was also assessed.

H. Barriers for spectacle use- To be filled by optometrist in all individuals not using any spectacles for vision correction and could not read N8 for near vision or the cause for distance presenting visual acuity less than 6/18 was refractive error.

2.5 SURVEY PREPARATION, SCHEDULE & DATA MANAGEMENT

2.5.1 Training and inter-observer agreement

The survey team comprised of one supervisor, one ophthalmologist, two optometrists, and four health workers. Prior to the survey, a two-day training was imparted to all team members regarding standardized study procedures, cluster selection & coding, enumeration methods, clinical examination and barrier information. Inter-observer agreement among the ophthalmologists for clinical diagnosis and among optometrists for distant and near visual acuity testing was performed in the hospital and field setting. Good inter-observer agreement was found for all survey procedures (kappa>0.8) among ophthalmologists and (kappa>0.9) among optometrists.

2.5.2 Data collection and examination

The filed survey was conducted during month of January 2013. Three survey teams, each team comprising of one supervisor, one ophthalmologist, two optometrists, and four health workers were deputed for data collection. The data was collected through a door-to-door survey from the selected clusters till 100 subjects were examined in each cluster. Distance visual acuity (VA) was measured with a Snellen ‘E’ chart with two 6/18 and 6/60 optotypes. If necessary, the distance between the subjects and the chart was decreased as per standard guideline to record visual acuity worse than 6/60. Aided and presenting visual acuity was recorded. If presenting visual acuity was <6/18, then pinhole measurement was done. VA of both eyes were recorded sequentially. Data were also collected about their spectacles uses, including where, and by whom they were tested & dispensed and at what price, patients’ satisfaction and, the barriers to correction of refractive errors.

In the field itself, the record forms were checked for completion. All the relevant sections on the record form are filed and signed with name and date by the optometrist and ophthalmologist of the concerned team.

2.3.6 IMPLEMENTATION OF SURVEY

Liasoning with Local Leadership

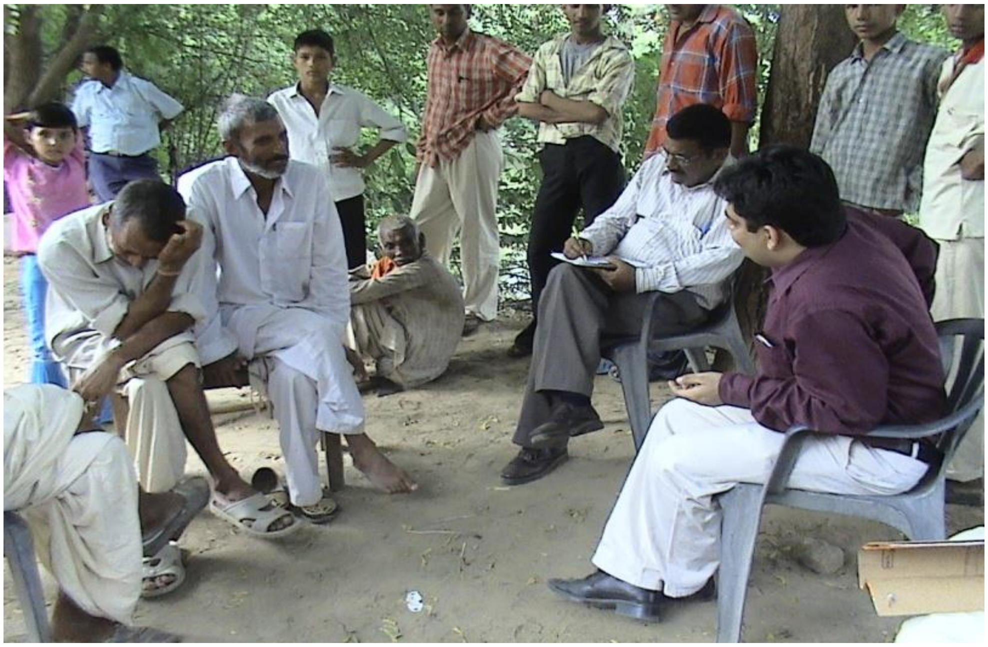

Mapping and selecting cluster boundaries

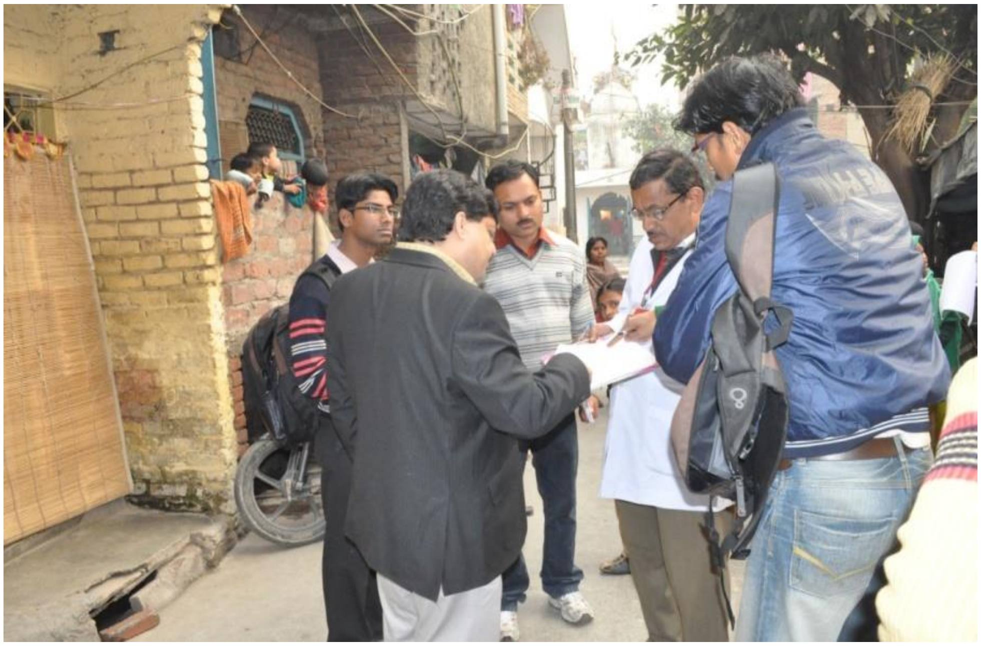

Mapping and selecting cluster boundaries

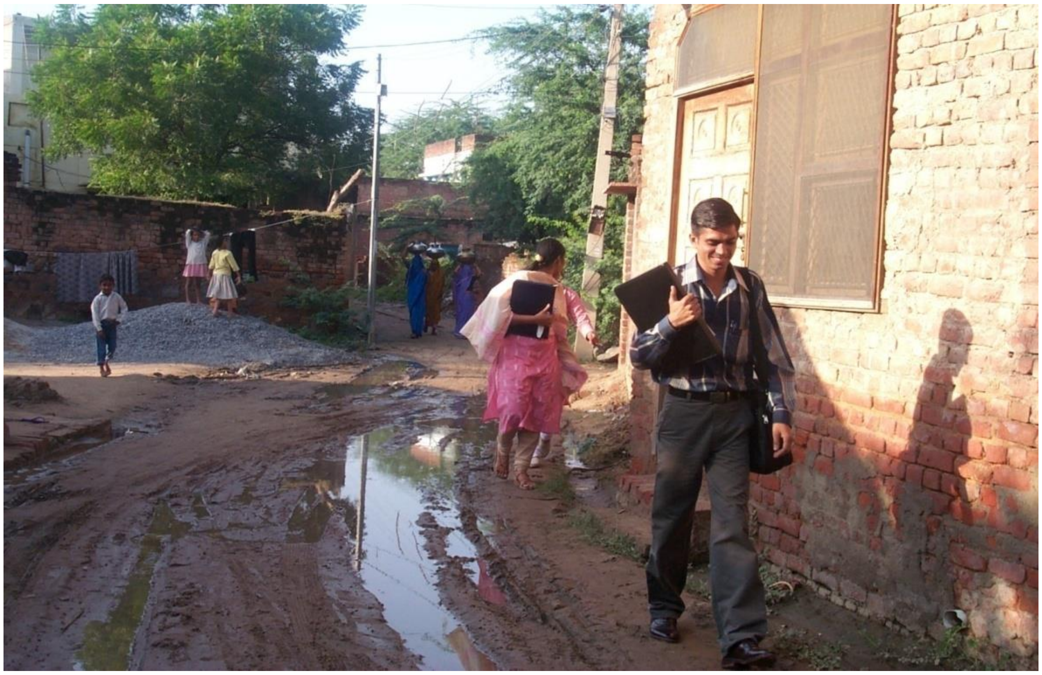

Motivation & Networking

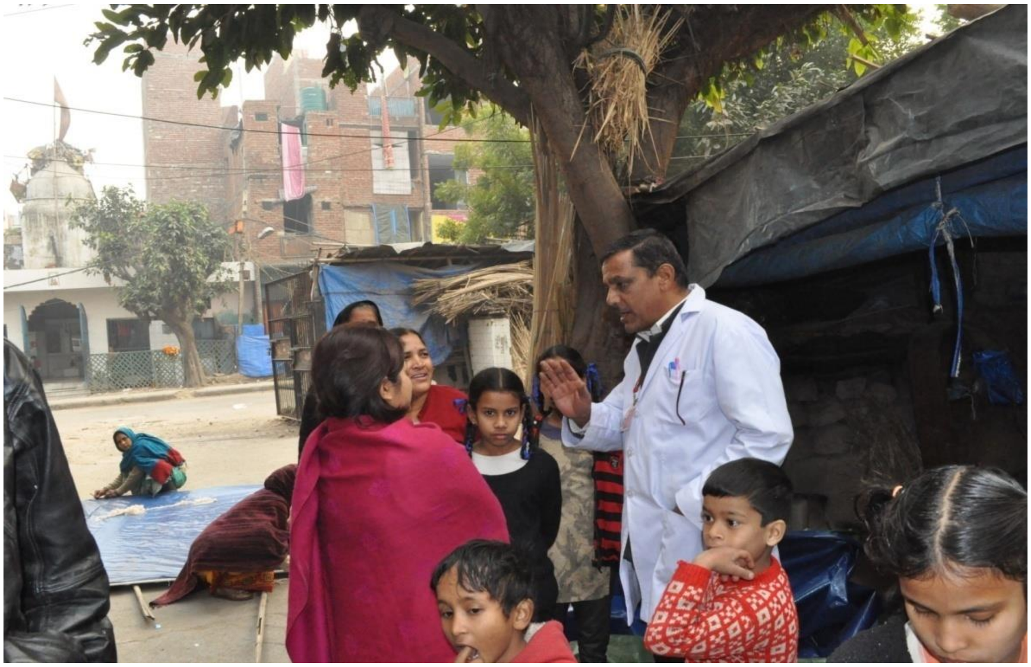

Enumeration of households in selected cluster

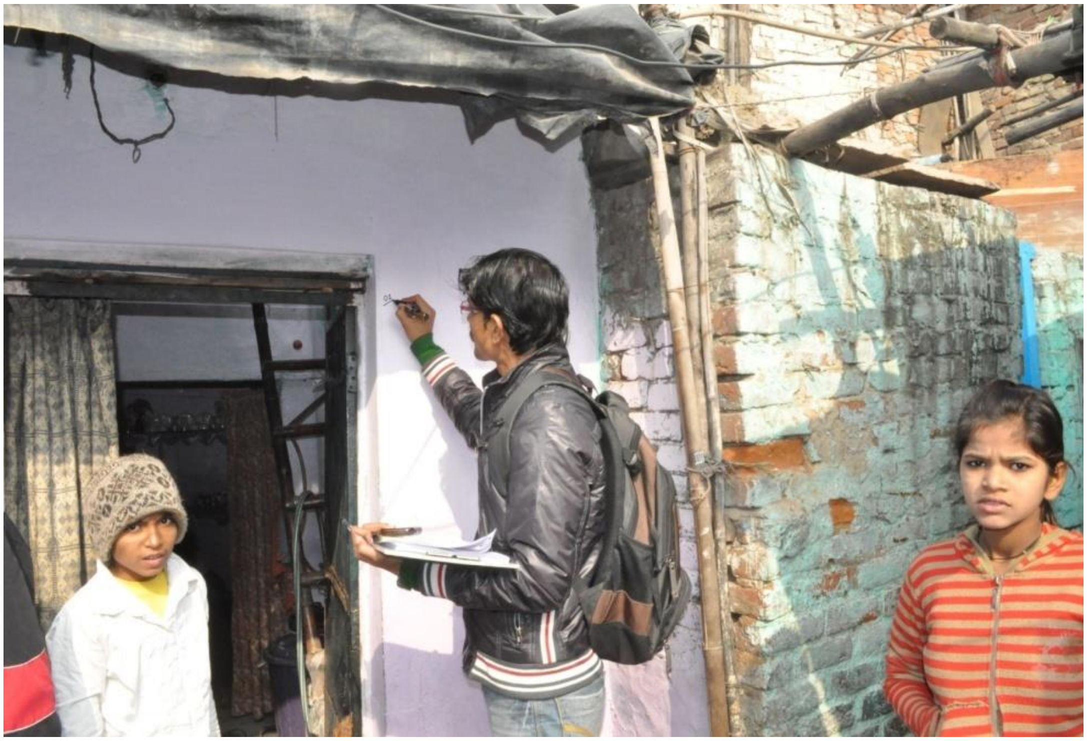

Written informed consent

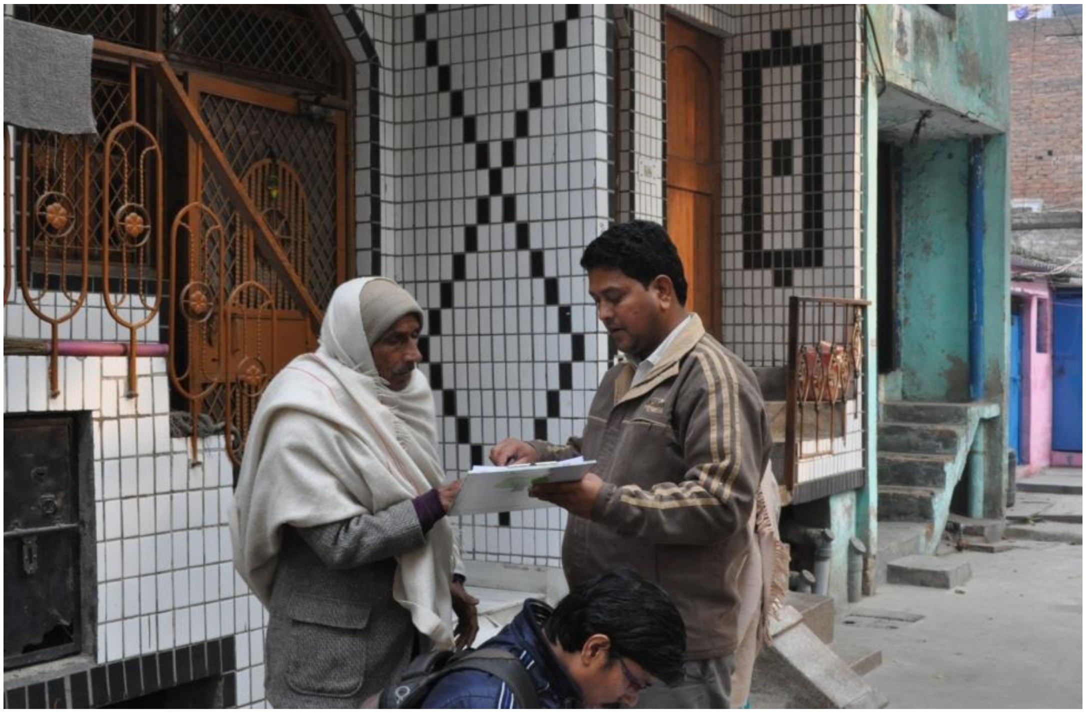

Recording demographic details of all eligible participants

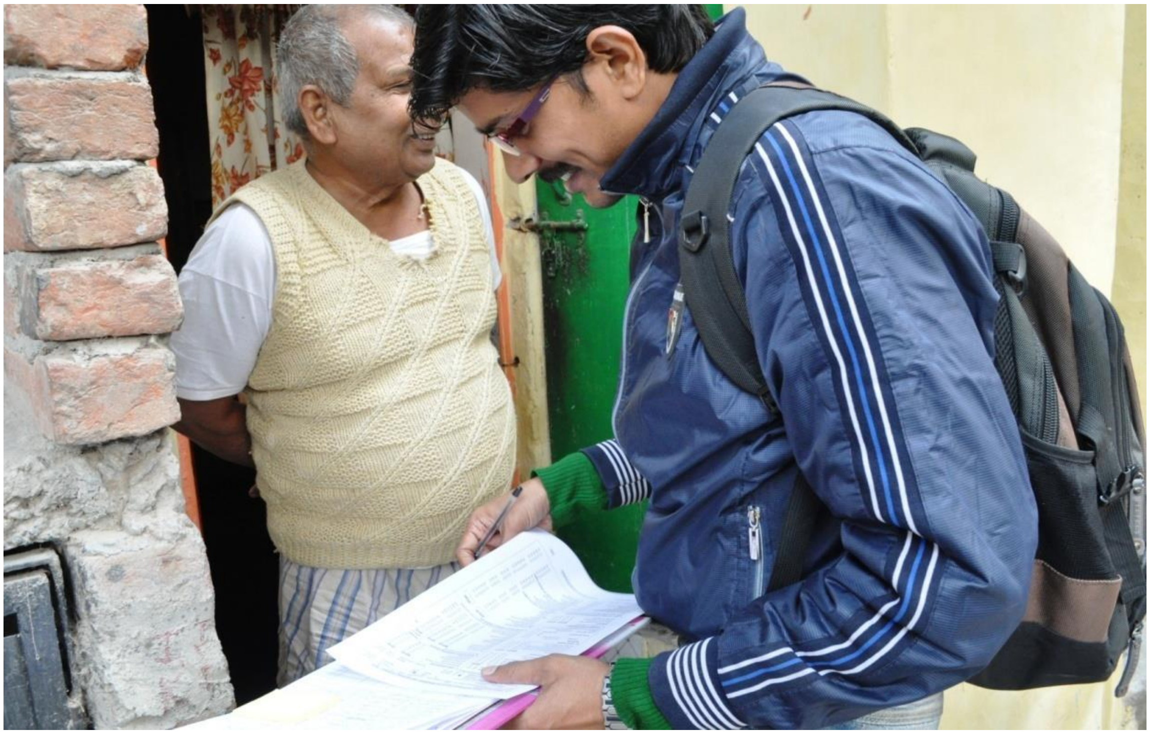

Measuring Visual Acuity for Distance with available correction

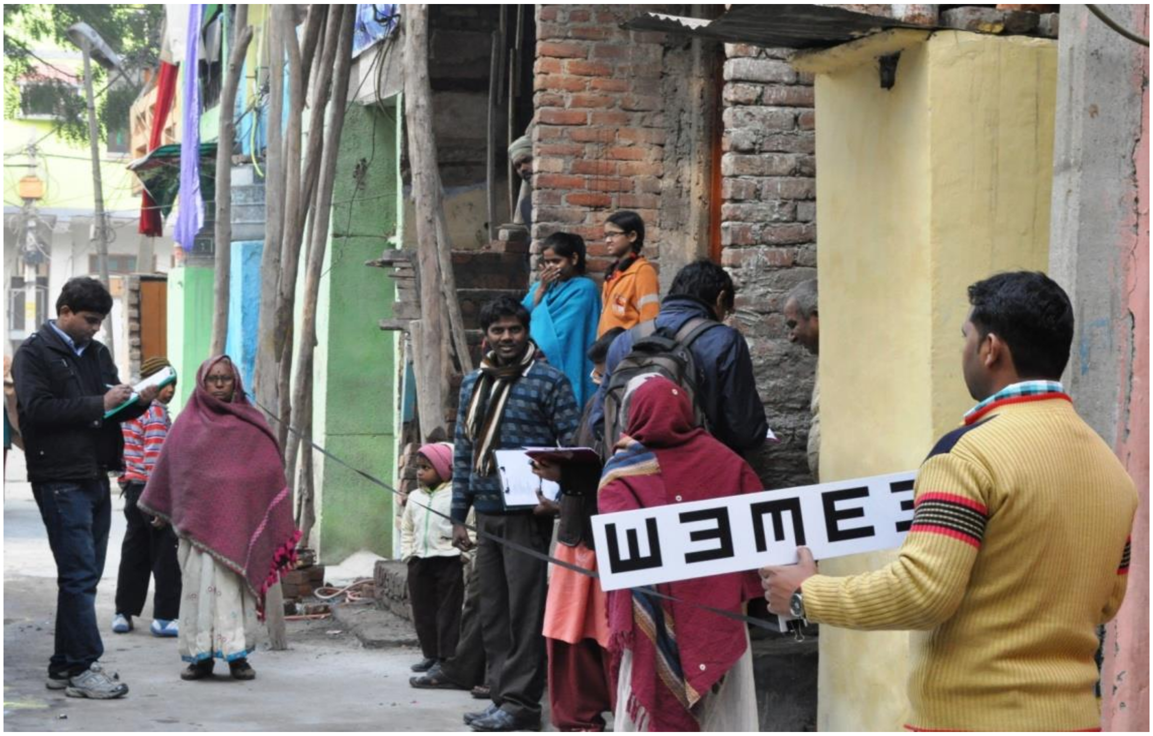

Measuring visual acuity with pin hole if Visual Acuity less than 6/18 in either eye

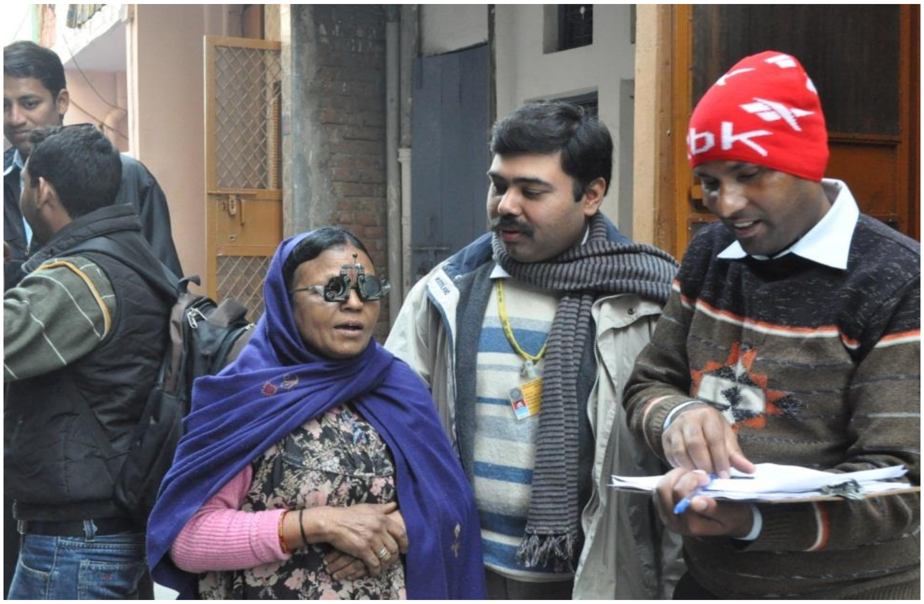

Near Vision Assessment

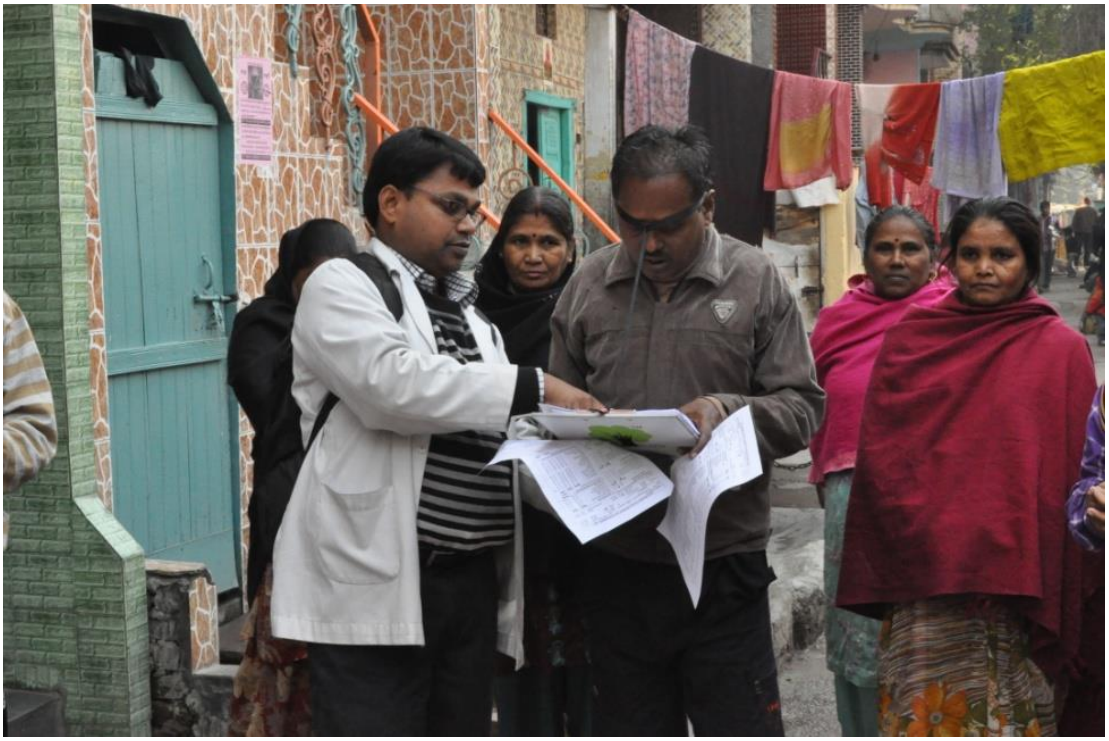

Near Vision Assessment

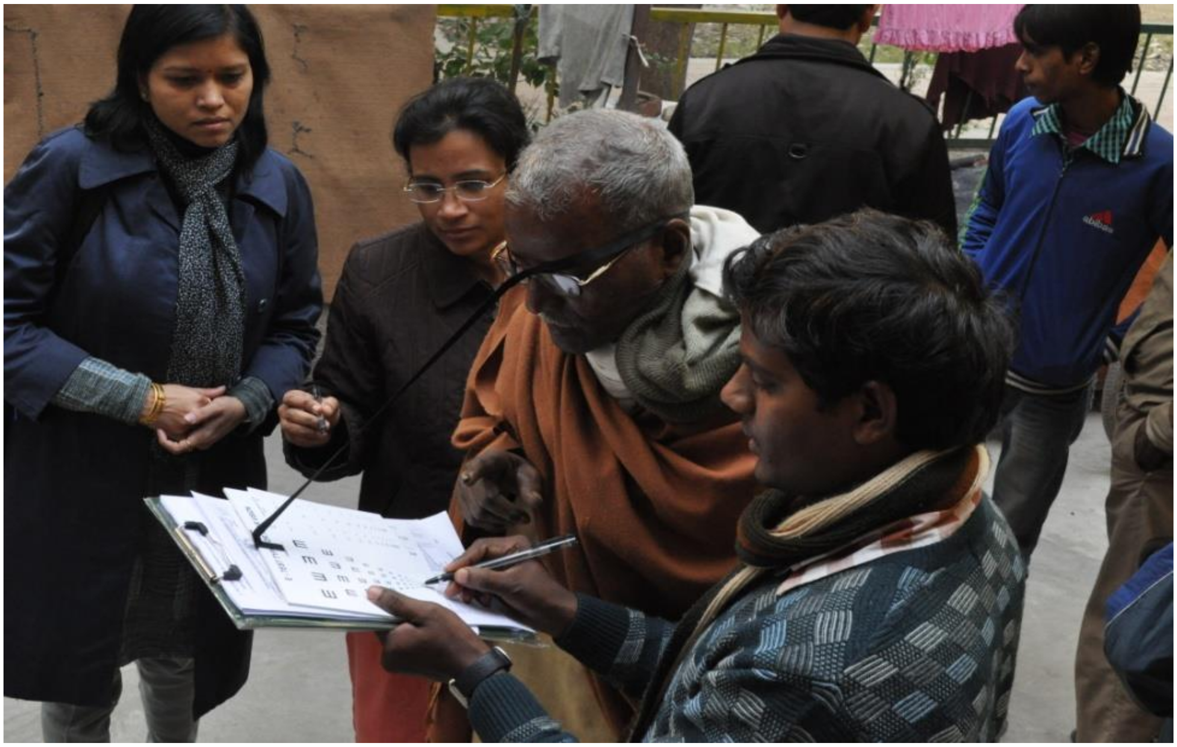

Torch light Examination

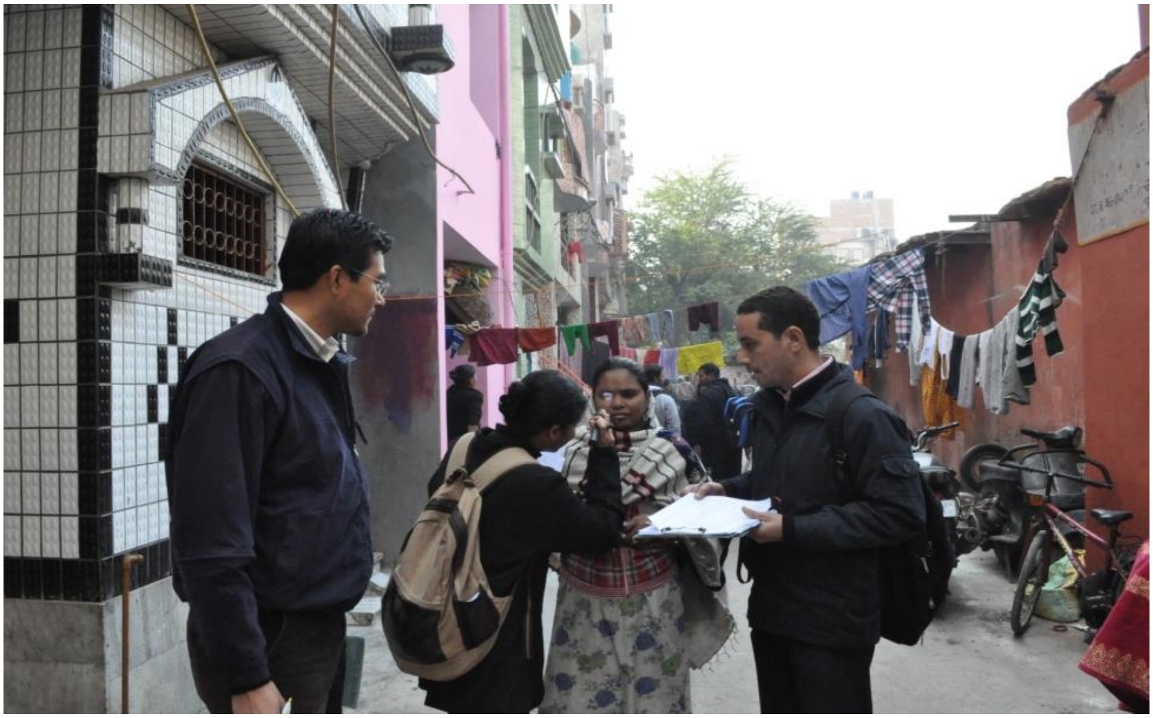

Slit lamp Examination

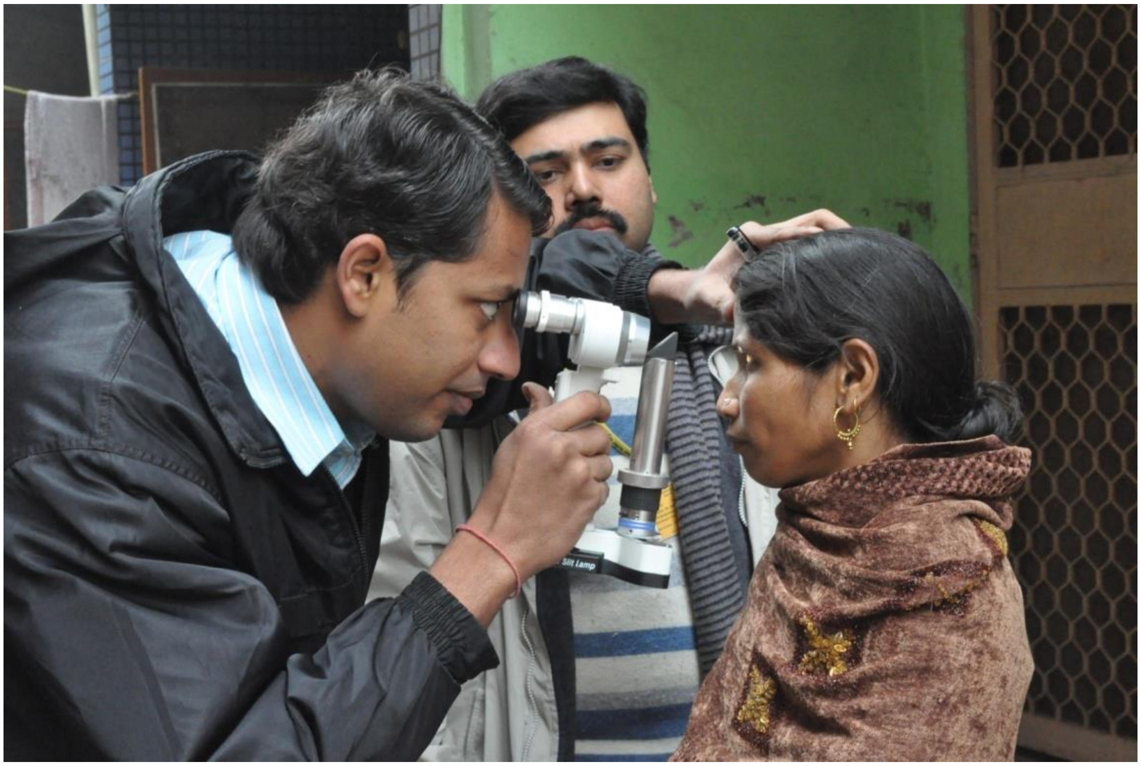

Direct ophthalmoscopy for retinal evaluation

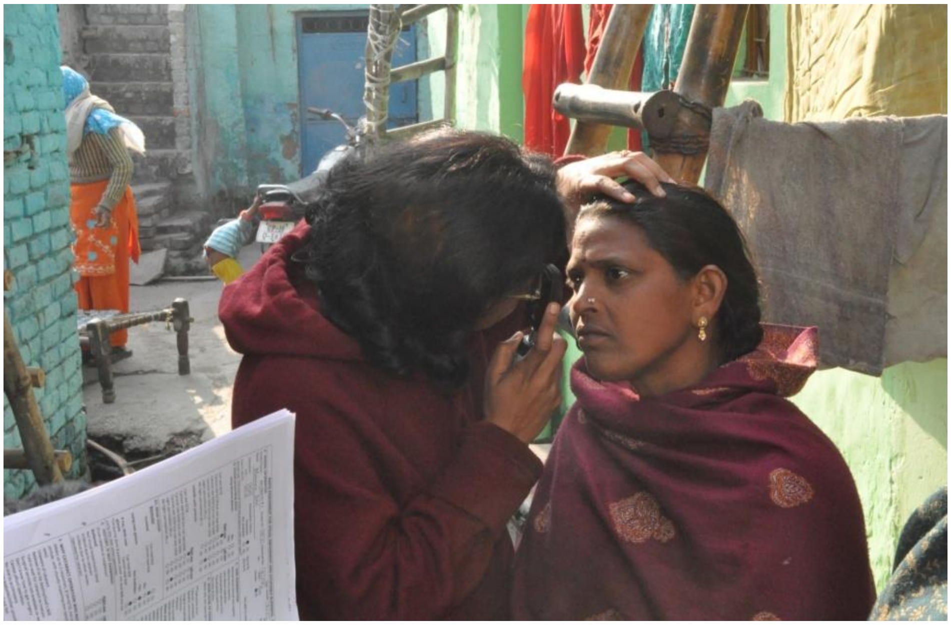

Filling questionnaires for spectacle use and Barriers questionnaire

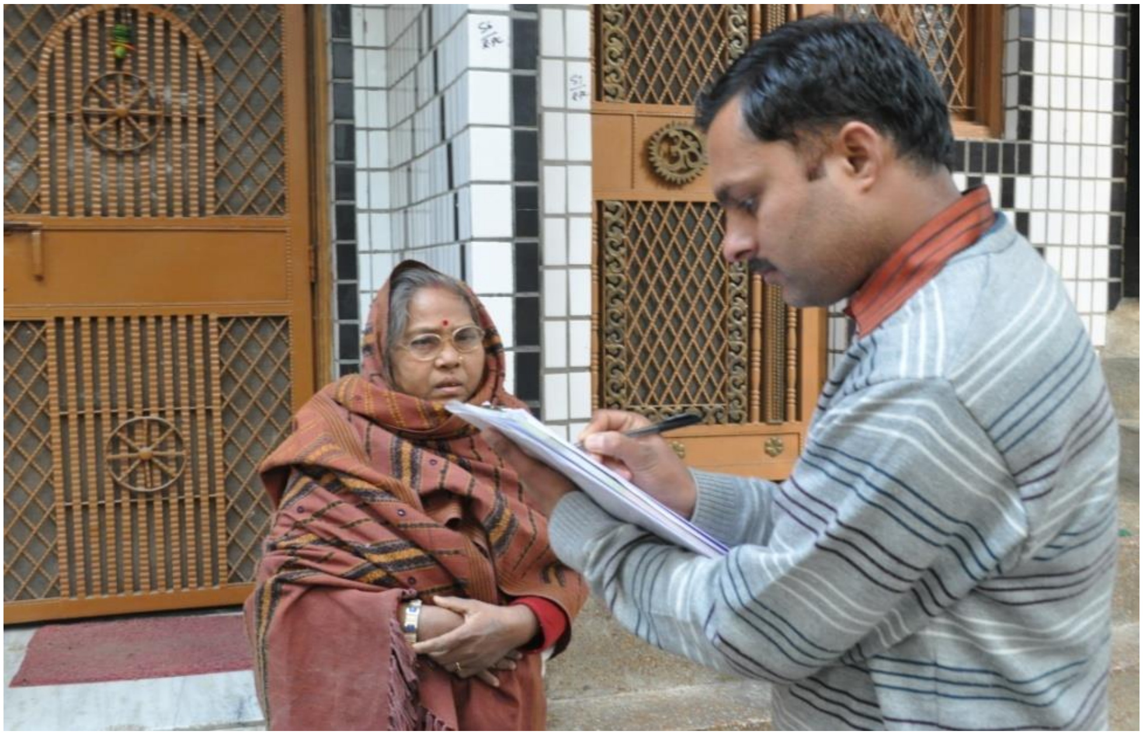

3. RESULTS

3.1 Basic Demographic Characteristics of the Survey Population

The Rapid Assessment of Visual impairment survey was conducted during January 2013 in the district of East Delhi. Overall, 2421 individuals aged 40 years and above were enumerated, of whom 2331 (96.3%) were examined. The response rate for examination was better among females (97.7%) than males (94.9%) (Table 1).Amongst the enumerated, 49.9% were females while 50.7 % of the examined study population were females (Table 1).

Table 1: Eligible Population and Response Rate of the RAVI survey

	Total		Examined		Not Examined		Coverage
	n	%	n	%	n	%	%
**Male**	1211	50.0	1149	49.3	62	68.9	94.9
**Female**	1210	49.9	1182	50.7	28	31.1	97.7
**Total**	2421	100.0	2331	96.3	90	3.7	96.3

Table 2: Age and Sex Distribution of the Examined Population

Age group (years)	Male		Female		Total	
	n	%	n	%	n	%
**40-49.**	468	40.7	552	46.7	1020	43.8
**50-59.**	298	25.9	260	22.0	558	23.9
**60-69**	279	24.3	269	22.8	548	23.5
**≥ 70**	104	9.1	101	8.5	205	8.8
**Total**	**1149**	**100.0**	**1182**	**100.0**	**2331**	**100.0**

Amongst the people examined, nearly half of the respondents (43.8%) were aged 40-49 years, and 8.8 % respondents were aged 70 years and above (Table 2). The males outnumbered the females in all age groups except in the 40-49 years age group.

The mean age of the respondents was 53.2 years (Range: 40-103 years) across the district. The mean age of the male participants was 53.9 years compared to 52.6 years amongst the females. Nearly half (48.5%) of the study population were either illiterate or could only read and write and had received no formal education. Only 3.5% individuals who were examined were graduates (Table 3).

Table 3: Educational Profile of the Examined Population						
**Educational Status**	**Male**		**Female**		Total	
	**N**	**%**	**n**	**%**	n	%
Illiterate	154	13.4	608	51.4	762	32.7
Can read & write	173	15.1	195	16.5	368	15.8
Primary	171	14.9	160	13.5	331	14.2
Middle/Secondary	578	50.3	210	17.8	788	33.8
Graduation & above	73	6.4	9	0.8	82	3.5
**Total**	**1,149**	**100**	**1,182**	**100**	**2,331**	**100**

3.2 Prevalence of Blindness and Visual Impairment

Based on bilateral presenting visual acuity in the better eye, 29 individuals were blind as per World Health Organization (WHO) definition (presenting visual acuity of <3/60 in the better eye), the prevalence being 1.24 % in the 40+ population (Table 4). As per the definition given by National Programme for Control of Blindness (NPCB), India (presenting visual acuity of <6/60 in the better eye), 51 people were blind with the prevalence of 2.2%. This includes people with both blindness (29) and severe visual impairment (22) as per WHO definition of blindness.

The prevalence of Moderate Visual Impairment (< 6/18 – 6/60 in the better eye) was 9.2 %.The prevalence of Severe Visual Impairment (Presenting Vision < 6/60 – 3/60 in the better eye) was 0.9% (Table 4).

The prevalence of unilateral blindness in the study population was 7.2 % (WHO definition) and 10.3% as per NPCB definition of blindness. Overall, visual impairment (defined as presenting visual acuity< 6/18 in the better eye) was seen in 266 individuals (11.4%).

Table: 4. Prevalence of Blindness, Severe Visual Impairment (SVI) and Moderate Visual Impairment (MVI)

Level of Visual impairment	Male (n=1149) n	%	Female N=1182) n	%	Total (n=2331) n	%
**Blindness-PVA<3/60 in the better eye**						
**Bilateral blindness**	9	0.8	20	1.7	**29**	1.2
**Unilateral blindness**	83	7.2	84	7.1	167	7.2
**SVI - PVA<6/60-3/60 in the better eye**						
**Bilateral SVI**	13	1.1	9	0.8	**22**	0.9
**Unilateral SVI**	37	3.2	48	4.1	85	3.6
**Blindness NPCB - PVA<6/60 in the better eye**						
**Bilateral blindness**	22	1.9	29	2.5	**51**	2.2
**Unilateral blindness**	112	9.7	128	10.8	240	10.3
**Moderate VI- PVA<6/18-6/60 in the better eye**						

**Bilateral MVI**	86	7.5	129	10.9	**215**	9.2
**All Visual impairment (VI) -PVA<6/18 in the better eye**						
**Bilateral VI**	108	9.4	158	13.4	**266**	11.4

PVA= Presenting Visual Acuity; VI=Visual Impairment

3.2.1 Association of Blindness and Visual Impairment with Gender

Using the NPCB cut-off, based on presenting vision, the prevalence of blindness among women was 1.32 times higher compared to men. The prevalence of blindness among males was 1.9% and among females, it was 2.5% (Table 4). Similarly, the prevalence of visual impairment (including blindness) was higher among females (13.4%) compared to males (10.9%).

3.2.2. Association of Blindness and Visual Impairment with Age

It was observed that the prevalence of blindness (presenting visual acuity < 6/60 in the better eye) increased with age and was noted to be maximum in people aged 70 years and above (Tables 5 & 6). The prevalence of NPCB blindness was 0.3% at 40-49 years of age and increased to 12.2% above the age of 70 years which is a 42-fold increase (Table 5). The maximum prevalence of blindness was seen in elderly females (14.9% as per national definition of blindness).

Table: 5. People with Bilateral Blindness (PVA<3/60 in Better Eye)

Age group (years)	Male (n=1149)		Female (n=1182)		Total (n=2331)	
	Total sample population n	People with bilateral blindness n %	Total sample population n	People with bilateral blindness n %	Total sample population n	People with bilateral blindness n %
**40-49**	468	2(0.4)	552	1(0.2)	1020	3(0.3)
**50-59**	298	0 (0.0)	260	1(0.4)	558	1(0.2)
**60-69**	279	4(1.4)	269	7(2.6)	548	11(2.0)
**≥ 70**	104	3(2.9)	101	11(10.9)	205	14(6.8)
**Total**	**1149**	**9(0.78)**	**1182**	**20(1.69)**	**2331**	**29(1.2)**

PVA= Presenting Visual Acuity

Table: 6. People with Bilateral Blindness as Per National Criteria (PVA<6/60 in Better Eye)						
	Male		Female		Total (n=2331)	
Age group (years)	Total sample population n	People with bilateral blindness n %	Total sample population n	People with bilateral blindness n %	Total sample population n	People with bilateral blindness n %
**40-49yrs.**	468	2(0.43)	552	1(.0.18)	1020	3(0.29)
**50-59yrs.**	298	6(2.01)	260	2(0.77)	558	8(1.43)
**60-69yrs.**	279	4(1.43)	269	11(4.09)	548	15 (2.73)
**≥ 70**	104	10 (9.61)	101	15 (14.85)	205	25 (12.2)
**Total**	**1149**	**22 (1.91)**	**1182**	**29 (2.45)**	**2331**	**51(2.19)**

PVA= Presenting Visual Acuity

It was observed that the prevalence of visual impairment (presenting visual acuity < 6/18 in the better eye) also increased with age. The prevalence was 2.4% at 40-49 years of age and increased to 39.0% above the age of 70 years (Table 7). The prevalence of visual impairment was maximum in both males and females in the elderly age group.

Table: 7. People with Visual Impairment (PVA<6/60 in Better Eye)						
	Male		Female		Total	
Age group (years)	n	VI (%)	n	VI (%)	n	VI (%)
**40-49**	468	10 (2.1)	552	14 (2.5)	1,020	24 (2.4)
**50-59**	298	21 (7.0)	260	33 (12.7)	558	54 (9.7)
**60-69**	279	44 (15.8)	269	64 (23.8)	548	108 (19.7)
**≥ 70**	104	33 (31.7)	101	47 (46.5)	205	80 (39.0)
**Total**	**1,149**	108 (9.4)	**1,182**	158 (13.4)	**2,331**	266 (11.4)

PVA= Presenting Visual Acuity; VI=Visual Impairment

3.3 Causes of Blindness & Visual Impairment

Cataract was the single largest cause of bilateral blindness (by WHO criteria) in this region (Table 8). Amongst the total blind population, 65.5% were blind due to cataract. Uncorrected refractive error was responsible for 10.3% of blindness. Trachoma and other corneal scarring was responsible for 6.9% of blindness. Posterior segment pathology was responsible for 10.3% of all bilateral blindness.

Considering NPCB criteria for definition of blindness, among 51 blind people, 37 (72.5 %) were blind due to cataract and 9.8% were blind due to uncorrected

refractive errors (Table 9).

When causes of severe visual impairment were analyzed, it was observed that cataract was responsible for 77.3% of severe visual impairment (presenting vision < <6/60 to 3/60 in the better eye) while uncorrected refractive errors were responsible for 13.6% (Table 10).

More than half of all the people with visual impairment (VI) were due to uncorrected refractive error. More than one-third of the people with VI had cataract (Table 11). Posterior segment disorders including diabetic retinopathy, corneal diseases, cataract surgical complications and uncorrected aphakia together were responsible for more than 10% of VI in this region.

Table 8: Principal Cause of Blindness (PVA<3/60 in Better Eye)

	Male		Female		Total	
	n	%	n	%	n	%
**Cataract, untreated**	6	66.7	13	65.0	19	65.5
**Uncorrected Refractive error**	2	22.2	1	5.0	3	10.3
**Aphakia, uncorrected**	0	0.0	1	5.0	1	3.4
**Surgical complications**	0	0.0	1	5.0	1	3.4
**Corneal Opacity**	0	0.0	2	10.0	2	6.9
**Posterior Segment Diseases**	1	11.1	2	10.0	3	10.3
**Total**	**9**	**100.0**	**20**	**100.0**	**29**	**100.0**

PVA= Presenting Visual Acuity

Table 9:. Principal cause of blindness (PVA<6/60 in better eye)

	Male		Female		Total	
	n	%	n	%	n	%
**Cataract, untreated**	16	72.7	21	72.4	37	72.5
**Uncorrected Refractive error**	3	13.6	2	6.9	5	9.8
**Aphakia, uncorrected**	0	0.0	1	3,4	1	1.9
**Surgical complications**	1	4.5	1	3.4	2	3.9
**Corneal Opacity**	0	0.0	2	6.9	2	3.9
**Other post. Segment/CNS**	2	9.1	2	6.9	4	7.8

Total	22	100.0	29	100.0	51	100.0

Table 10: Principal causes of Severe Visual Impairment (P VA<6/60-3/60 in better eye)

	Male		Female		Total	
	n	%	n	%	n	%
Uncorrected Refractive error	2	15.4	1	11.1	3	13.6
Cataract, untreated	9	69.2	8	88.9	17	77.3
Surgical complications	1	7.7	0	0.0	1	4.5
Other post. Segment/CNS	1	7.7	0	0.0	1	4.5
**Total**	**13**	**100.0**	**9**	**100.0**	**22**	**100.0**

PVA= Presenting Visual Acuity

Table 11: Principal causes of Visual Impairment (P VA<6/18 in better eye)

	Male		Female		Total	
	n	%	n	%	n	%
Uncorrected Refractive error	60	55.6	82	51.9	142	53.4
Cataract, untreated	35	32.4	55	34.8	90	33.8
Aphakia, uncorrected	1	0.9	2	1.3	3	1.1
Surgical complication	5	4.6	4	2.5	9	3.4
Phthisis	0	0.0	1	0.6	1	0.4
Corneal Opacity	0	0.0	4	2.5	4	1.5
Glaucoma	2	1.9	0	0.0	2	0.8
Diabetic retinopathy	1	0.9	4	2.5	5	1.9
ARMD	0	0.0	2	1.3	2	0.8
Posterior Segment Diseases	4	3.7	4	2.5	8	3.0
**Total**	**108**	**100.0**	**158**	**100.0**	**266**	**100.0**

PVA= Presenting Visual Acuity

If we consider the total number of blind eyes (presenting visual acuity less than 3/60 in any eye), the main causes of blindness in these 225 eyes (Table 12)

were cataract (55.6%), posterior segment diseases (17.8%) and corneal diseases (7.6%).

Table 12: Main cause of blindness in Blind Eyes: P VA<3/60 in any eye

	Male		Female		Total	
	**N**	**%**	**n**	**%**	**n**	**%**
**Refractive error**	1	1.0	2	1.6	3	1.3
**Cataract, untreated**	55	54.5	70	56.5	125	55.6
**Aphakia, uncorrected**	0	0.0	5	4.0	5	2.2
**Surgical complications**	2	2.0	5	4.0	7	3.1
**Phthisis**	4	4.0	3	2.4	7	3.1
**Other corneal scar**	7	6.9	10	8.1	17	7.6
**Globe abnormality**	3	3.0	2	1.6	5	2.2
**Glaucoma**	3	3.0	2	1.6	5	2.2
**Diabetic retinopathy**	3	3.0	5	4.0	8	3.6
**ARMD**	2	2.0	1	0.8	3	1.3
**Other post. Segment/CNS**	21	20.8	19	15.3	40	17.8
**Total**	**101**	**100.0**	**124**	**100.0**	**225**	**100.0**

PVA= Presenting Visual Acuity

A total of 578 people had visual impairment (PVA<6/18) in any eye. The distribution of visual acuity in these 578 people with visual impairment in any eye has been demonstrated in Table 13. When visual acuity was assessed in each eye, a total of 844 eyes of 578 people had visual acuity of less than 6/18 with available correction. The main cause of visually impaired eyes was refractive error (42.2%) followed by cataract (35.5%). The main cause of blindness in these 884 eyes was cataract and posterior segment diseases (Table 14).

Table: 13. Distribution of Visual Acuity in Patients with Visual Impairment in any eye (n=578)

	Male(n=264)		Female(n=314)		Total(n=578)	
Presenting Vision	**n**	**%**	**n**	**%**	**n**	**%**
<3/60	92	34.8	104	33.1	196	33.9
<6/60-3/60	42	15.9	53	16.9	95	16.4

<6/18-6/60	130	49.2	157	50.0	287	49.7
Total	264	100.0	314	100.0	578	100.0

Table 14: Principal causes of Visual Impairment (Eyes)

	VA <6/18(%)	VA <6/60(%)	VA 3/60(%)
Refractive error	356(42.2)	15(04.4)	3(11.3)
Cataract, untreated	300(35.5)	203(59.4)	125(55.6)
Aphakia, uncorrected	7(00.8)	7(02.0)	5(02.2)
Surgical complication	30(03.5)	14(04.1)	7(03.1)
Phthisis	7(00.8)	7(02.0)	7(03.1)
Corneal Opacity	26(03.1)	19(05.6)	17(07.6)
Globe abnormality	5(00.6)	5(01.5)	5(02.2)
Glaucoma	10(01.2)	6(01.7)	5(02.2)
Diabetic retinopathy	20(02.4)	10(02.9)	8(03.6)
ARMD*	16(02.0)	6(01.7)	30(1.3)
Posterior Segment Diseases	65(07.7)	48(14.0)	38(16.9)
Others	2(00.2)	2(00.6)	2(00.9)
Total	844(100)	342(100)	225(100)

*Age Related Macular Degeneration; VA= Visual Acuity

3.4 Refractive Error as a Cause of Visual Impairment

Uncorrected Refractive error was the principal cause of visual impairment in 53.4% of the study population in this area. Uncorrected refractive error was present in 356 eyes of 275 (11.8%) people aged 40 years and older. The distribution of presenting visual acuity in 356 eyes with refractive error is tabulated in Table 15.

Out of a total of 2331 individuals who were examined, 468 (20.1%) were using spectacles for distance. Out of these, 314 (67.1%) were using bifocal correction while the rest were using spectacles for distance vision only. Hence, the spectacle coverage was 20.1%, although 52 people using spectacles still had an uncorrected refractive error.

Table 15: Visual Acuity in Eyes with Refractive Error (n=356)

Presenting Vision	Male(n=154) N	%	Female(n=202) N	%	Total(n=356) n	%
<3/60	1	0.6	2	1.0	3	0.8
<6/60-3/60	4	2.6	8	4.0	12	3.4
<6/18-6/60	149	96.8	192	95.0	341	95.8
Total	154	100.0	202	100.0	356	100.0

3.4.1 Use of spectacles for distance correction

The details of type of spectacles, duration of spectacle wear, place where prescribed and procured, and the cost of the glasses was noted for all 468 participants using glasses for distance vision (Table 16). It was noted that the majority of people using spectacles were satisfied (83.3 %), were wearing spectacles for more than five years (45.7 %), had been prescribed (59.0%) and supplied (88.3%) with the spectacles from a private source. Nearly half (50.2%) of the study participants using distance correction, had bought the spectacles for 201 to 500 Indian Rupees.

Table 16: Spectacle wear characteristics in the study population using spectacles for distance correction

**Duration of Spectacle Wear**		
**Time Duration (years)**	**n**	**%**
<1	62	13.3
1-5	192	41.0
5-10	96	20.5
>10	118	25.2
Total	468	100.0

**Distribution of Bifocal and Unifocal Spectacles**		
**Type of Spectacles used**	**n**	**%**
For Distance	154	32.91
Both(Near & Distance)	314	67.09
Total	468	100

**Source of Refractive Error Services**		
Health facility	**N**	**%**
Private eye specialist	227	48.5
Government Dispensary/PHC	33	7.1
Government hospital	118	25.2

NGO hospital	18	3.9
Spectacles shop	49	10.5
Eye camp	15	3.2
Miscellaneous	8	1.7
Total	468	100.0

**Source of Spectacles**		
Place of procurement	n	%
Spectacle shop/Private Doctor	413	88.3
Government/NGO hospital	38	8.1
Miscellaneous	17	3.6
Total	468	100.0

**Cost of Spectacles**		
Cost (INR)	n	%
Free of cost	21	4.5
00-200	76	16.2
201-500	235	50.2
>501	132	28.2
Others ?	4	0.9
Total	468	100.0

**Satisfaction with spectacles**		
Response	n	%
Yes	390	83.3
No	78	16.7
Total	468	100.0

3.4.2 Barriers to use of spectacles for distant vision

In 275 participants with uncorrected refractive error for distance, barriers were asked from 233 individuals (Table 17) as 52 people were already using spectacles, but were under-corrected. Out of the 233 individuals, perceptible barriers were only received from 191 participants. Hence, data was not available for 38 individuals who did not give any categorical reason for not wearing spectacles in spite of visual impairment due to refractive error.

Table 17: Barriers to use of spectacles for distant vision

	N	%
Unaware about visual impairment in ones eye	9	4.7
Financial reason	28	14.7
Need not felt	52	27.2
Unaware of place where refractive error services provided	5	2.6
Personal reasons (friends make fun)	20	10.5
Time less/less priority to eyes	22	11.5
Spectacles not comfortable/ no improvement of vision with spectacles	27	14.1
Other	27	14.1
Other treatment (yoga, naturopathy)	1	0.5
Total	191	100.0

3.5 Prevalence of Presbyopia and spectacle coverage

A total of 798 (34.2 %) participants were presbyopic (those who could not read N8 binocularly unaided) in this study population. Females outnumbered the males in all age groups with presbyopia. The prevalence was maximum in the fifth decade (40-49 years) in both males and females. Most of the female presbyopes were illiterate while the males who were presbyopes were largely educated up to eighth or twelfth standard (Table 18).

Table 18: Socio-demographic Parameters of Participants with Presbyopia (N=798)

	Male		Female		Total	
**Age (years)**	**N**	**%**	**n**	**%**	**n**	**%**
**40-49**	103	32.7	166	34.37	269	33.71
**50-59**	98	31.11	133	27.54	231	28.95
**60-69**	79	25.08	122	25.26	201	25.19
**≥70**	35	11.11	62	12.84	97	12.16
**Total**	315	100	483	100	798	100
**Education**						
**Illiterate**	63	20	283	58.59	346	43.36
**can read & write**	39	12.38	52	10.77	91	11.4
**Primary**	49	15.56	65	13.46	114	14.29

**Middle/Secondary**	144	45.71	80	16.56	224	28.07
**Graduation & above**	20	6.35	3	0.62	23	2.88
**Total**	315	100	483	100	798	100

Overall, 796 of 2331 (34.1%) participants were using presbyopic glasses in the study population. The comparative age, gender and education profile of individuals using and those not using presbyopic spectacles is shown in Table 18. The proportion of males using presbyopic spectacles was higher than those not using near glasses. The literacy level of those wearing presbyopic spectacles was higher than those not wearing spectacles for near vision (Table 19).

**Table 19: Socio-demographic Parameters of Participants using near vision spectacles (N=796)**						
	**Yes**		**No**		**Total**	
**Age (years)**	**n**	**%**	**N**	**%**	**n**	**%**
**40-49**	317	39.82	703	45.8	1,020	43.76
**50-59**	246	30.9	312	20.33	558	23.94
**60-69**	182	22.86	366	23.84	548	23.51
**≥70**	51	6.41	154	10.03	205	8.79
**Total**	796	100	1,535	100	2,331	100.00
**Gender**						
**Male**	440	55.28	709	46.19	1149	49.29
**Female**	356	44.72	826	53.81	1182	50.71
**Total**	796	100	1535	100	2331	100
**Education**						
**Illiterate**	165	20.73	597	38.89	762	32.69
**Can read & write**	111	13.94	257	16.74	368	15.79
**Primary**	134	16.83	197	12.83	331	14.2
**Middle/Secondary**	341	42.84	447	29.12	788	33.81
**Graduation & above**	45	5.65	37	2.41	82	3.52
**Total**	796	100	1,535	100	2,331	100

Out of these 796 participants, only 397 were found to be presbyopic by our study definition (those who could not read N8 binocularly unaided). Nearly half (50.1%) of the study population was using presbyopic glasses for uniocular near vision correction or for reading N6 notation on the near vision chart.

Out of the 397 presbyopes (who could not read N8 binocularly unaided), 371 could read N8 binocularly with their available correction. Twenty six presbyopes could not read N8 binocularly with their current spectacles. Thus the **'met need'** in this study population was 46.5% (371/798).

3.5.1 Use of spectacles for near correction

The age and gender distribution with their education profile of participants wearing presbyopic correction is shown in Table 20. Most of the near spectacle users were in the age group of 40-49 years.

Table 20: Socio-demographic Parameters of Presbyopic Participants using near vision spectacles (N=397)

Age (years)	Male		Female		Total	
	n	%	n	%	n	%
40-49	66	35.7	86	40.6	152	38.3
50-59	69	37.3	63	29.7	132	33.3
60-69	35	18.9	51	24.1	86	21.7
≥70	15	8.1	12	5.7	27	6.8
Total	185	100.0	212	100.0	397	100.0
**Education**	**Male**		**Female**		**Total**	
illiterate	16	8.7	85	40.1	101	25.4
can read & write	21	11.4	31	14.6	52	13.1
Primary	28	15.1	45	21.2	73	18.4
Middle/Secondary	101	54.6	48	22.6	149	37.5
Graduation & above	19	10.3	3	1.4	22	5.5
Total	185	100.0	212	100.0	397	100.0

The details of type of presbyopic spectacles, duration of spectacle wear, place where the spectacles were prescribed and procured, and the cost of the glasses was noted for all 397 participants using glasses for distance vision (Table 21). It was noted that the majority of people using spectacles were satisfied (80.9 %), were wearing spectacles for less than five years (60.5 %), had been prescribed (65.5%) and supplied (81.9%) with the spectacles from a private source. Nearly half (47.1 %) of the study participants using distance correction, had bought the spectacles for 201 to 500 Indian Rupees.

Table 21: Spectacle wear characteristics in the study population using spectacles for near correction

**Duration of Spectacle Wear for near correction**		
**Time Duration (years)**	**n**	**%**
<1	50	12.6
1-4	190	47.9
5-10	82	20.7
>10	75	18.9
Total	397	100.0
**Distribution of Bifocal and Near Spectacles**		
**Type of Spectacles used**	**n**	**%**
For Near	225	56.7
Both(Near & Distance)	172	43.3
Total	397	100.0
**Place where presbyopic correction obtained**		
Health facility	**N**	**%**
Private eye specialist	161	40.6
Government Dispensary/PHC	21	5.3
Government hospital	87	21.9
NGO hospital	15	3.8
Spectacles shop	59	14.9
Eye camp	38	9.6
Miscellaneous	16	4.0
Total	397	100.0
**Source of Spectacles**		
Place of procurement	n	%
Spectacle shop/Private Doctor	325	81.9
Government/NGO hospital	27	6.8
Miscellaneous	45	11.3
Total	397	100.0
**Cost of Spectacles for near vision**		
Cost (INR)	n	%
Free of cost	18	4.5
0-200	118	29.7
201-500	187	47.1
>501	68	17.1
Others ?	6	1.5
Total	397	100.0

Satisfaction with spectacles for near vision		
Response	n	%
Yes	321	80.9
No	76	19.1
Total	397	100.0

3.5.2 Barriers to use of spectacles for near vision

In 798 participants with presbyopia, barriers could be recorded for 364 or 401 eligible 233 individuals (Table 22) as 397 people were already using spectacles. Out of the 401 individuals who were not using spectacles inspite of problems in near vision, data was not available for 37 participants. The major barriers recorded were that the near vision correction was not required in their routine lives and for some cost of spectacles was an issue.

Table 22: Barriers to use of spectacles for presbyopia (n=364)

	n	%
Unaware about visual impairment in ones eye	10	2.7
Financial reason	73	19.4
Need not felt	94	24.9
Unaware of place where refractive error services provided	6	1.6
Personal reasons (friends make fun)	31	8.2
Time less/less priority to eyes	39	10.3
Spectacles uncomfortable/ no improvement of vision with spectacles	67	17.8
Other	54	14.3
Other treatment (yoga, naturopathy)	3	0.8
Total	377*	100.0

*More than one barrier reported in 364 participants

3.5.3 Unmet Need for Presbyopia

In participants with unmet need (defined as those study participants who were presbyopic but were not wearing any spectacles for near vision), the age and gender distribution is shown in Table 23. Most of these participants were illiterate, females and aged 60 years and above.

Table 23: Socio-demographic Parameters of Presbyopic Participants not using near vision spectacles: Unmet Need (N=397)						
**Age (years)**	**Male**		**Female**		**Total**	
	n	%	n	%	n	%
40-49	37	26.2	81	28.3	118	27.6
50-59	33	23.4	72	25.2	105	24.6
60-69	47	33.3	77	26.9	124	29.0
≥70	24	17.0	56	19.6	80	18.7
Total	141	100.0	286	100.0	427	100.0
**Education**	**Male**		**Female**		**Total**	
Illiterate	49	34.8	209	73.1	258	60.4
can read & write	20	14.2	23	8.0	43	10.1
Primary	22	15.6	21	7.3	43	10.1n
Middle/Secondary	48	34.0	33	11.5	81	19.0
graduation & above	2	1.4	0	0.0	2	0.5
Total	141	100.0	286	100.0	427	100.0

3.6 Comparison of Presenting and Pinhole Vision

The National Program for Control of Blindness defines individuals with a vision less than 6/60 in the better eye as blind. Using this cut off, the prevalence of blindness was observed to be 2.2% among the 40+ population, based on presenting vision (Table 24). Using pinhole vision, the prevalence of blindness was 1.3%.

Even though best correction was not done as part of the survey, all individuals with a presenting vision < 6/18 in any eye were examined with a pinhole. It was observed that with a pinhole, nearly one-third (31.03%) individuals with blindness could improve with pinhole (Table 26). The maximum improvement with pin hole was seen in participants with moderate visual impairment (54 vs 245)

Table 24: Prevalence of blindness, severe visual impairment (SVI) and visual impairment (VI) by pinhole- VA

Level of Visual acuity	Male(n=1149)		Female(n=1182)		Total (n=2331)	
	n	%	n	%	n	%
**Blindness-VA<3/60 in the better eye with PinholeVA**						
**Bilateral blindness**	4	0.3	16	1.4	20	0.9
**Unilateral blindness**	74	6.4	66	5.6	140	6.0
**Blind eyes**	82	3.6	98	4.1	180	3.9

**SVI - VA<6/60-3/60 in the better eye with Pinhole VA**						
**Bilateral blindness SVI**	4	0.3	6	0.5	10	0.4
**Unilateral blindness**	16	1.4	21	1.8	37	1.6
**Eyes with SVI**	24	1.0	33	1.4	57	2.4
**NPCB blind - VA<6/60 in the better eye with Pinhole VA**						
**Bilateral NPCB blindness**	8	0.7	22	1.9	30	1.3
**Unilateral NPCB blindness**	87	7.6	84	7.1	171	7.3
**NPCB Blind eyes**	103	4.5	106	4.5	209	4.5
**Moderate VI - VA<6/18-6/60 in the better eye with Pinhole VA**						
**Bilateral MVI**	21	1.8	34	2.9	55	2.3
**Visual Impairment- VA<6/18 in the better eye, with Pinhole VA**						
**Bilateral VI**	49	4.2	74	6.3	123	5.3

PVA= Presenting Visual Acuity

3.7 Prevalence of Cataract and related blindness and visual impairment

The prevalence of cataract blindness in this study population (aged 40 years and above) was 0.56% (CI: 0.26, 0.86). It was more in females [0.68% (CI: 0.21, 1.14)] than males [0.44% (CI: 0.05, 0.82)]. The prevalence of unilateral blindness was 4.55% (CI: 3.70, 5.39) as per the WHO definition of blindness.

A total of 301 eyes with visual impairment had cataract and 209 eyes were blind as per NPCB definition, due to cataract (Table 25). There were more females than males with cataract in the study population. The age and gender distribution of people (and eyes) with cataract leading to unilateral and bilateral blindness and visual impairment are tabulated in Tables 26 to 34.

Table 25: Magnitude of cataract blindness (as per national and international definition) and visual impairment with cataract

	Male(1149)		Female(1182)		Total(2331)	
	n	%	n	%	n	%
**Cataract Blindness-PVA<3/60 in the better eye**						
**Bilateral cataract blind**	5	0.4	8	0.7	13	0.6
**Unilateral cataract blind**	50	4.4	56	4.7	106	4.5
Cataract blind eyes	60	5.2	72	6.1	132	5.7
**Cataract Blindness NPCB- PVA<6/60 in the better eye**						
**Bilateral cataract blind**	13	1.1	15	1.3	28	1.2
**Unilateral cataract blind**	70	6.1	83	7.0	153	6.6

Cataract blind eyes	96	8.4	113	9.6	209	9.0
**Cataract with VI- PVA<6/18 in the better eye**						
**Bilateral VI due to cataract**	26	2.3	38	3.2	64	2.7
**Unilateral VI due to cataract**	78	6.8	95	8.0	173	7.4
**VI eyes with cataract**	130	11.3	171	14.5	301	12.9

PVA= Presenting Visual Acuity;

VI=Visual Impairment

Table 26: Age and Gender distribution of people with Bilateral Blindness (PVA<3/60 in better eye) due to cataract

	Male		Female		Total	
Age group (years)	**n**	**%**	**n**	**%**	**n**	**%**
40-49	1	20.0	0	0.0	1	7.7
50-59	0	0.0	1	12.5	1	7.7
60-69	2	40.0	3	37.5	5	38.5
≥70	2	40.0	4	50.0	6	46.1
Total	**5**	**100.0**	**8**	**100.0**	**13**	**100.0**

PVA= Presenting Visual Acuity

Table 27: Age and Gender distribution of people with Unilateral Blindness (PVA<3/60 in better eye) due to cataract

	Male		Female		Total	
Age group (years)	**n**	**%**	**n**	**%**	**n**	**%**
40-49	4	8.0	5	8.9	9	8.5
50-59	10	20.0	7	12.5	17	16.0
60-69	17	34.0	28	50.0	45	42.5
≥70	19	38.0	16	28.6	35	33.0
Total	**50**	**100.0**	**56**	**100.0**	**106**	**100.0**

PVA= Presenting Visual Acuity

Table 28: Age and Gender distribution of people with Blind eyes (PVA<3/60 in any eye) due to cataract

	Male		Female		Total	
Age group (years)	**n**	**%**	**n**	**%**	**n**	**%**
40-49	6	10.0	5	6.9	11	8.3
50-59	10	16.7	9	12.5	19	14.4

60-69	21	35.0	34	47.2	55	41.7
≥70	23	38.3	24	33.3	47	35.6
Total	**60**	**100.0**	**72**	**100.0**	**132**	**100.0**

PVA= Presenting Visual Acuity

Table 29: Age and Gender distribution of people with Bilateral Blindness (PVA<6/60 in better eye) due to cataract

	Male		Female		Total	
Age group (years)	**n**	**%**	**n**	**%**	**n**	**%**
40-49	1	7.7	0	0.0	1	3.6
50-59	4	30.8	1	6.7	5	17.9
60-69	2	15.4	7	46.7	9	32.1
≥70	6	46.2	7	46.7	13	46.4
Total	**13**	**100.0**	**15**	**100.0**	**28**	**100.0**

PVA= Presenting Visual Acuity

Table 30: Age and Gender distribution of people with Unilateral Blindness (PVA<6/60 in better eye) due to cataract

	Male		Female		Total	
Age group (years)	**n**	**%**	**n**	**%**	**n**	**%**
40-49	5	7.1	7	8.4	12	7.8
50-59	16	22.9	18	21.7	34	22.2
60-69	28	40.0	38	45.8	66	43.1
≥70	21	30.0	20	24.1	41	26.8
Total	**70**	**100.0**	**83**	**100.0**	**153**	**100.0**

Table 31: Age and Gender distribution of people with Blind Eyes (PVA<6/60 in any eye) due to cataract

	Male		Female		Total	
Age group (years)	**n**	**%**	**n**	**%**	**n**	**%**
40-49	7	7.3	7	6.2	14	12.4
50-59	24	25.0	20	17.7	44	38.9
60-69	32	33.3	52	46.0	84	74.3
≥70	33	34.4	34	30.1	67	59.3
Total	**96**	**100.0**	**113**	**100.0**	**209**	**100.0**

PVA= Presenting Visual Acuity

Table 32: Age and Gender distribution of people with Bilateral Visual Impairment (PVA<6/18 in better eye) due to cataract

Age group (years)	Male n	%	Female n	%	Total n	%
40-49	3	11.5	2	5.3	5	7.8
50-59	5	19.2	5	13.2	10	15.6
60-69	8	30.8	16	42.1	24	37.5
≥70	10	38.5	15	39.5	25	39.1
Total	**26**	**100.0**	**38**	**100.0**	**64**	**100.0**

PVA= Presenting Visual Acuity

Table 33: Age and Gender distribution of people with Unilateral Visual Impairment (PVA<6/18 in any eye) due to cataract

Age group (years)	Male n	%	Female n	%	Total n	%
40-49	6	7.7	5	5.3	11	6.4
50-59	16	20.5	18	18.9	34	19.7
60-69	34	43.6	47	49.5	81	46.8
≥70	22	28.2	25	26.3	47	27.2
Total	**78**	**100.0**	**95**	**100.0**	**173**	**100.0**

PVA= Presenting Visual Acuity

Table 34: Age and Gender distribution of people with Visually Impaired eyes (PVA<6/18 in any eye) due to cataract

Age group (years)	Male n	%	Female n	%	Total n	%
40-49	12	9.2	9	5.3	21	7.0
50-59	26	20.0	28	16.4	54	17.9
60-69	50	38.5	79	46.2	129	42.9
≥70	42	32.3	55	32.2	97	32.2
Total	**130**	**100.0**	**171**	**100.0**	**301**	**100.0**

PVA= Presenting Visual Acuity

3.8 Cataract Operated participants
3.8.1 Profile of Cataract Operated Individuals

A total of 460 cataract surgeries (Table 35) were reported among 304 people from the study population. More females reported surgery (163) compared to males (141). It was observed that the maximum numbers of unilateral and bilateral cataract surgeries were performed in people aged 60 years and above (Tables 36 & 37). Out of all the pseudophakic eyes (460) in 304 individuals, more than three- fourth of the surgeries were done in individuals who were 60 years and older (Table 38). Out of a total of 460 eyes that underwent cataract surgery, 156 people were bilaterally operated while 148 participants had undergone cataract surgery in one eye only.

Table 35: Gender distribution of unilateral and bilateral aphakia and pseudophakia

	Male(1149)		Female(1182)		Total(2331)	
	**N**	**%**	**n**	**%**	**n**	**%**
Bilateral (pseudo)aphakia	69	6.0	87	7.4	156	6.7
unilateral (pseudo)aphakia	72	6.3	76	6.4	148	6.3
(pseudo)aphakia eyes	210	18.3	250	21.2	460	19.7

Table 36: Age Distribution of People with Bilateral (Pseudo)Aphakia

	**Male**		**Female**		**Total**	
**Age group (years)**	**n**	**%**	**n**	**n**	**%**	**N**
**40-49**	2	2.9	10	11.5	12	7.7
**50-59**	11	15.9	12	13.8	23	14.7
**60-69**	29	42.0	37	42.5	66	42.3
**≥70**	27	39.1	28	32.2	55	35.3
Total	**69**	**100.0**	**87**	**100.0**	**156**	**100.0**

Table 37:Age Distribution of People With Unilateral (Pseudo)Aphakia)

	**Male**		**Female**		**Total**	
**Age group (years)**	**n**	**%**	**n**	**n**	**%**	**N**
**40-49**	6	8.3	7	9.2	13	8.8
**50-59**	19	26.4	7	9.2	26	17.6
**60-69**	25	34.7	37	48.7	62	41.9
**≥70**	22	30.6	25	32.9	47	31.8
**Total**	**72**	**100.0**	**76**	**100.0**	**148**	**100.0**

Table 38: Characteristics of Pseudophakic eyes

	Male		Female		Total	
**Age group (years)**	**N**	**%**	**n**	**n**	**%**	**N**
**40-49**	10	4.8	27	10.8	37	8.0
**50-59**	41	19.5	31	12.4	72	15.7
**60-69**	83	39.5	111	44.4	194	42.2
**≥70**	76	36.2	81	32.4	157	34.1
Total	**210**	**100.0**	**250**	**100.0**	**460**	**100.0**

3.8.2 Characteristics of Cataract Surgery

Out of a total of 460 cataract surgeries in the study population, details about the type of surgery, place and cost of surgery was available for 457 eyes only. Interestingly, females underwent more non-IOL surgeries than males (Table 39).

Table 39: Proportion and type of surgery (eyes wise)

	Male		Female		Total	
	**n**	**%**	**n**	**%**	**n**	**%**
**Non-IOL**	8	3.9	30	12.1	38	8.3
**IOL-implant**	200	96.2	219	87.9	419	91.7
**Total**	**208**	**100**	**249**	**100**	**457**	**100**

The highest numbers of surgeries were done in the age group 50-74 years amongst both males and females. The maximum proportion of cataract surgeries was done in the 50-59 years age group (42.3%) amongst both males and females in participants with either unilateral or bilateral aphakia/pseudophakia (Tables 40 & 41). Amongst the males, 96.2% surgeries were done with an IOL implant compared to 87.9% among the females. The male-female differentials were significant for type of surgery in this study population (Table 40 & 41).

Table 40: Age at time of surgery & type of surgery in male participants

	Non-IOL		IOL-implant		Total	
	**Eyes**	**%**	**Eyes**	**%**	**Eyes**	**%**
**30-39yrs.**	0	0.0	6	3.0	6	2.9
**40-44yrs.**	0	0.0	7	3.5	7	3.4
**45-49yrs.**	0	0.0	8	4.0	8	3.8
**50-54yrs.**	2	25.0	30	15.0	32	15.4
**55-59yrs.**	3	37.5	53	26.5	56	26.9
**60-64yrs.**	1	12.5	47	23.5	48	23.1

**65-69yrs.**	1	12.5	25	12.5	26	12.5
**70-74yrs.**	0	0.0	15	7.5	15	7.2
**75-79yrs.**	1	12.5	5	2.5	6	2.9
**≥80**	0	0.0	4	2.0	4	1.9
**Total**	**8**	**100.0**	**200**	**100.0**	**208**	**100.0**

Table 41: Age at time of surgery & type of surgery in female participants

	Non-IOL		IOL-implant			
	Eyes	%	Eyes	%	Eyes	%
**30-39yrs.**	1	3.3	12	5.5	13	5.2
**40-44yrs.**	3	10.0	15	6.8	18	7.2
**45-49yrs.**	2	6.7	21	9.6	23	9.2
**50-54yrs.**	3	10.0	25	11.4	28	11.2
**55-59yrs.**	1	3.3	44	20.1	45	18.1
**60-64yrs.**	6	20.0	53	24.2	59	23.7
**65-69yrs.**	8	26.7	29	13.2	37	14.9
**70-74yrs.**	5	16.7	13	5.9	18	7.2
**75-79yrs.**	1	3.3	5	2.3	6	2.4
**≥80**	0	0.0	2	0.9	2	0.8
**Total**	**30**	**100.0**	**219**	**100.0**	**249**	**100.0**

3.8.3 Visual Outcome after Surgery

Visual acuity after surgery was analyzed separately for non IOL and IOL surgery.

The proportion of surgeries with an IOL implant was 91.7% (Table 39).When no IOL was used, 28.9% had a vision better than 6/18 in the operated eye while 42.1% had vision less than 6/60 (Table 42). Based on presenting vision, 28.9% of the operated eyes had moderate visual impairment in the operated eye.

With IOL surgery, 93.5% had vision better than 6/60 in the operated eye while only 6.4% had vision less than 6/60 (Table 42). There was significant difference in visual outcome after IOL surgery compared to non-IOL surgery.

Table 42 : Visual acuity of operated eyes in sample with Presenting VA

	Non-IOL		IOL-implant		Total	
	Eyes	%	Eyes	%	Eyes	%
**Can See 6/18**	11	28.9	328	78.3	339	74.2
**Cannot See 6/18, Can See 6/60**	11	28.9	64	15.3	75	16.4
**Cannot See 6/60**	16	42.1	27	6.4	43	9.4

Total	**38**	**100.0**	**419**	**100.0**	**457**	**100.0**

There were more surgeries in the last 5 years(239) which was responsible for 52.2% of all surgeries reported (Table 43). As mortality increases with age, it is logical that most surgeries would be reported by survivors (the most recently operated).The IOL surgery rate was only 86.2% in surgeries reported before 2008 compared to 96.6% among surgeries in the last 5 years (Table 43 & 44). Pseudophakia had better visual outcome than aphakia regardless of the year and time when surgery was performed (Tables 43 & 44).

The major causes of poor visual outcome in aphakes was refractive error in recent surgeries (done within last 3 years) (Table 45) and surgical complications, ocular co-morbidity and refractive error in old surgeries (Table 46). Moreover, in cataract surgeries with IOL implant, the important causes of poor visual outcome was ocular co-morbidity, refractive error,surgical complications and posterior capsular opacification in recent surgeries (done within last 3 years) (Table 20) and refractive error and posterior capsular opacification in old surgeries (Table 46).

Table 43: Visual acuity with available correction in eyes operated less than 5 years age - with Presenting VA

	Non-IOL		IOL-implant		Total	
	**Eyes**	**%**	**Eyes**	**%**	**Eyes**	**%**
**Can See 6/18**	1	12.5	190	82.3	191	80.3
**Cannot See 6/18, Can See 6/60**	2	25.0	29	12.6	31	13.0
**Cannot See 6/60**	5	62.5	11	4.8	17	6.7
Total	**8**	**100.0**	**230**	**100.0**	**238**	**100.0**

Table 44: Visual acuity with available correction in eyes operated 5 or more than 5 years age - Presenting VA

	Non-IOL		IOL-implant		Couching		Total	
	**Eyes**	**%**	**Eyes**	**%**	**Eyes**	**%**	**Eyes**	**%**
**Can See 6/18**	10	33.3	138	73.4	0	0.0	148	67.9
**Cannot See 6/18, Can See 6/60**	9	30.0	35	18.6	0	0.0	44	20.2
**Cannot See 6/60**	11	36.7	15	8.0	0	0.0	26	11.9
Total	**30**	**100.0**	**188**	**100.0**	**0**	**0.0**	**218**	**100.0**

Table 45: Post-op presenting VA and cause outcome in eyes operated less than 3 years ago

	**Selection**	**Surgery**	**Spectacles**	**Long term**	**Does not**

							complications		apply	
Non-IOL	**Eyes**	**%**	**Eyes**	**%**	**Eyes**	**%**	**Eyes**	**%**	**Eyes**	**%**
**Can See 6/18**	0	0.0	0	0.0	0	0.0	0	0.0	1	100.0
**Cannot See 6/18, Can See 6/60**	0	0.0	0	0.0	1	50.0	0	0.0	0	0.0
**Cannot See 6/60**	0	0.0	0	0.0	1	50.0	1	100.0	0	0.0
Total	**0**	**0.0**	**0**	**0.0**	**2**	**100.0**	**1**	**0.0**	**1**	**100.0**
IOL implant										
**Can See 6/18**	0	0.0	0	0.0	0	0.0	0	0.0	118	95.2
**Cannot See 6/18, Can See 6/60**	7	53.8	1	50.0	7	70.0	0	0.0	6	4.8
**Cannot See 6/60**	6	46.2	1	50.0	3	30.0	2	100.0	0	0.0
Total	**13**	**100.0**	**2**	**100.0**	**10**	**100.0**	**2**	**100.0**	**124**	**100.0**

Table 46: Post-op presenting VA and cause outcome in eyes operated 3 or more than 3 years ago

	**Selection**		**Surgery**		**Spectacles**		**Long term complications**		**Does not apply**	
Non-IOL	**Eyes**	**%**	**Eyes**	**%**	**Eyes**	**%**	**Eyes**	**%**	**Eyes**	**%**
**Can See 6/18**	0	0.0	0	0.0	0	0.0	0	0.0	10	90.9
**Cannot See 6/18, Can See 6/60**	6	60.0	2	22.2	1	25.0	0	0.0	1	9.1
**Cannot See 6/60**	4	40.0	7	77.8	3	75.0	0	0.0	0	0.0
Total	**10**	**100.0**	**9**	**100.0**	**4**	**100.0**	**0**	**0.0**	**11**	**100.0**
IOL implant										
**Can See 6/18**	0	0.0	0	0.0	0	0.0	0	0.0	210	99.1
**Cannot See 6/18, Can See 6/60**	6	50.0	6	60.0	19	100.0	10	66.7	2	0.9
**Cannot See 6/60**	6	50.0	4	40.0	0	0.0	5	33.3	0	0.0
Total	**12**	**100.0**	**10**	**100.0**	**19**	**100.0**	**15**	**100.0**	**212**	**100.0**

3.8.4 Place of Surgery

The proportion of cataract surgeries performed in make-shift camps or outreach locations is very low (Table 47). More than a quarter of all surgeries were performed at private

facilities while more than half were at Government facilities (Table 47). Amongst aphakes, the surgeries done in the government set up had poorer visual outcome (Table 48). Nearly three-fourth of all the cataract surgeries with intraocular lens implant had better visual outcome in both the government and private set up.

Table 47: Place of cataract surgery

	Male		Female		Total	
	**n**	**%**	**n**	**%**	**n**	**%**
Government. hospital	115	55.3	145	58.2	260	56.9
Voluntary/Charitable hospital	27	13.0	17	6.8	44	9.6
Private hospital	63	30.3	81	32.5	144	31.5
Eye camp	1	0.5	6	2.4	7	1.5
Traditional setting	2	1.0	0	0.0	2	0.4
Total	208	100.0	249	100.0	457	100.0

Table 48: Post-op VA(Presenting VA) with available correction by place of surgery

	**Govt. hospital**		**Voluntary hospital**		**Private hospital**		**Eye camp**		**Traditional**	
Non-IOL	**Eyes**	**%**	**Eyes**	**%**	**Eyes**	**%**	**Eyes**	**%**	**Eyes**	**%**
**Can See 6/18**	6	22.2	2	66.7	3	60.0	0	0.0	0	0.0
**Cannot See 6/18, Can See 6/60**	9	33.3	0	0.0	0	0.0	2	66.7	0	0.0
**Cannot See 6/60**	12	44.4	1	33.3	2	40.0	1	33.3	0	0.0
Total	**27**	**100.0**	**3**	**100.0**	**5**	**100.0**	**3**	**100.0**	**0**	**0.0**
IOL implant										
**Can See 6/18**	170	73.0	30	73.2	122	87.8	4	100.0	2	100.0
**Cannot See 6/18, Can See 6/60**	41	17.6	8	19.5	15	10.8	0	0.0	0	0.0
**Cannot See 6/60**	22	9.4	3	7.3	2	1.4	0	0.0	0	0.0
Total	**233**	**100.0**	**41**	**100.0**	**139**	**100.0**	**4**	**100.0**	**2**	**100.0**

3.8.5 Satisfaction after cataract surgery

The participants were also enquired about satisfaction after cataract surgery and males appeared to be more satisfied than females. Nearly 10% were not satisfied with cataract surgery (Table 49). When the level of satisfaction was compared with the type and visual outcome after cataract

surgery, then a similar proportion was satisfied and dissatisfied amongst aphakes (Table 50). Among pseudophakes, a large proportion showed a high level of satisfaction (91.6%). As expected, better visual outcome was associated with a higher level of satisfaction.

Table 49: Are you satisfied with results of cataract surgery? (Eyes wise)

	Male		Female		Total	
	n	%	n	%	n	%
**Very satisfied**	160	76.9	172	69.1	332	72.6
**Partially satisfied**	27	13.0	45	18.1	72	15.8
**Indifferent**	3	1.4	3	1.2	6	1.3
**Partially dissatisfied**	12	5.8	15	6.0	27	5.9
**Very dissatisfied**	6	2.9	14	5.6	20	4.4
Total	**208**	**100.0**	**249**	**100.0**	**457**	**100.0**

Table 50: Post-op presenting VA and satisfaction with results of cataract surgery? (Eyes wise)

	Very satisfied		Partially satisfied		Indifferent		Part. dissatisfied		Very dissatisfied	
Non-IOL	Eyes	(%)	Eyes	%	Eyes	%	Eyes	%	Eyes	%
**Can See 6/18**	5	55.6	5	45.5	0	0.0	0	0.0	1	10.0
**Cannot See 6/18, Can See 6/60**	3	33.3	5	45.5	0	0.0	2	25.0	1	10.0
**Cannot See 6/60**	1	11.1	1	9.1	0	0.0	6	75.0	8	80.0
Total	**9**	**100.0**	**11**	**100.0**	**0**	**0.0**	**8**	**100.0**	**10**	**100.0**
IOL implant										
**Can See 6/18**	282	87.3	38	62.3	0	0.0	6	31.6	2	20.0
**Cannot See 6/18, Can See 6/60**	38	11.8	16	26.2	1	16.7	7	36.8	2	20.0
**Cannot See 6/60**	3	0.9	7	11.5	5	83.3	6	31.6	6	60.0
Total	**323**	**100.0**	**61**	**100.0**	**6**	**100.0**	**19**	**100.0**	**10**	**100.0**

3.9 Cataract Surgical Coverage (Persons)

The cataract surgical coverage among persons was calculated as follows:

Coverage (Persons) = <u>No. of persons operated in one/both eyes</u> x 100

No. of persons operated + No. of unoperated cataract blind/VI persons

The cataract surgical rates was calculated separately for cataract blind persons with presenting vision < 3/60 in the better eye, for persons with presenting vision < 6/60 in the better eye and for persons with visual impairment.

Using presenting vision < 3/60 to define the cataract blind persons, 81.2% of persons needing cataract surgery were covered by surgery (Table 11) while using presenting vision < 6/60 to define the cataract blind, 74.5 % of persons had one or both eyes operated. Among the people with Moderate visual impairment (presenting visual acuity less than 6/18 in the better eye), cataract surgical coverage was (65.9%)

Table 51: Cataract Surgical Coverage

	Male			Female			Total		
	**n**	**%**	**N**	**n**	**%**	**N**	**n**	**%**	**N**
Cataract Surgical Coverage (persons) - percentage									
VA<3/60	141	96.6	146	163	93.7	174	304	95.0	320
VA<6/60	141	90.4	156	163	90.1	181	304	90.2	337
VA<6/18	141	82.9	170	163	78.4	208	304	80.4	378
Cataract Surgical Coverage (eyes) - percentage									
VA<3/60	210	79.2	265	250	78.1	320	460	78.6	585
VA<6/60	210	69.3	303	250	69.4	360	460	69.4	663
VA<6/18	210	61.8	340	250	59.4	421	460	60.4	761

3.12 Barriers to Cataract Surgery

The barriers to cataract surgery among the cataract blind (presenting vision <3/60 in the better eye) were also studied. The barriers were categorized as awareness related, service related and other barriers. The main reasons were affordability and that they were unaware of their cataract (Table 52). Fear was stated by 8.6% while similar proportion reported having systemic co-morbidity like uncontrolled hypertension, diabetes mellitus, and pulmonary disease contra-indicating cataract surgery. Similar barriers were reported when participants with cataract with different categories of visual impairment (bilateral

and unilateral blind, NPCB blind, SVI and visual impairment) and this has been elaborated in Tables 52 to 59. Lack of escorts, adequate vision in the fellow eye and lack of time were other barriers reported.

REASON FOR CATARACT BLIND PEOPLE NOT AVAILING CATARACT SURGICAL SERVICES

Table 52: Barriers to Cataract Surgery in bilateral blind due to cataract

	Male		Female		Total	
Barriers	**n**	**%**	**n**	**%**	**n**	**%**
**Unaware of cataract**	3	42.9	1	6.3	4	17.4
**Told to wait for cataract to mature**	0	0.0	1	6.3	1	4.3
**Surgical services not available or very**	0	0.0	1	6.3	1	4.3
**Cannot afford operation**	0	0.0	4	25.0	4	17.4
**No one to accompany**	0	0.0	4	25.0	4	17.4
**No time available/other priorities**	0	0.0	1	6.3	1	4.3
**Old age and need not felt**	1	14.3	0	0.0	1	4.3
**Fear of operation**	0	0.0	1	6.3	1	4.3
**Fear of losing eye sight**	0	0.0	1	6.3	1	4.3
**Other disease contra-indicating operation**	3	42.9	1	6.3	4	17.4
**Other**	0	0.0	1	6.3	1	4.3
Total	**7**	**100.0**	**16**	**100.0**	**23**	**100.0**

Table 53: Barriers to Cataract Surgery in bilateral NPCB blind due to cataract

	Male		Female		Total	
Barriers	**n**	**%**	**n**	**%**	**n**	**%**
**Unaware of cataract**	6	37.5	3	11.1	9	20.9
**Told to wait for cataract to mature**	0	0.0	1	3.7	1	2.3
**Surgical services not available or very**	0	0.0	1	3.7	1	2.3
**Don't know how to get surgery**	0	0.0	2	7.4	2	4.7
**Cannot afford operation**	0	0.0	6	22.2	6	14.0
**No one to accompany**	1	6.3	5	18.5	6	14.0
**No time available/other priorities**	1	6.3	2	7.4	3	7.0
**Old age and need not felt**	2	12.5	0	0.0	2	4.7
**One eye adequate vision/need not felt**	1	6.3	0	0.0	1	2.3
**Fear of operation**	0	0.0	3	11.1	3	7.0
**Fear of loosing eye sight**	0	0.0	2	7.4	2	4.7
**Other disease contra-indicating operation**	5	31.3	1	3.7	6	14.0
**Other**	0	0.0	1	3.7	1	2.3

Total	**16**	**100.0**	**27**	**100.0**	**43**	**100.0**

Table 54: Barriers to Cataract Surgery in participants with visual impairment (PVA<6/18) due to cataract

	Male		Female		Total	
Barriers	**n**	**%**	**n**	**%**	**n**	**%**
**Unaware of cataract**	13	39.4	14	24.6	27	30.0
**Told to wait for cataract to mature**	0	0.0	4	7.0	4	4.4
**Surgical services not available or very**	0	0.0	1	1.8	1	1.1
**Don't know how to get surgery**	0	0.0	3	5.3	3	3.3
**Cannot afford operation**	4	12.1	8	14.0	12	13.3
**No one to accompany**	2	6.1	8	14.0	10	11.1
**No time available/other priorities**	2	6.1	4	7.0	6	6.7
**Old age and need not felt**	3	9.1	3	5.3	6	6.7
**One eye adequate vision/need not felt**	1	3.0	0	0.0	1	1.1
**Fear of operation**	0	0.0	5	8.8	5	5.6
**Fear of loosing eye sight**	1	3.0	3	5.3	4	4.4
**Other disease contra-indicating operation**	7	21.2	3	5.3	10	11.1
**Other**	0	0.0	1	1.8	1	1.1
**Total**	33	100.0	57	100.0	90	100.0

Table 55: Barriers to Cataract Surgery in unilateral blind due to cataract

	Male		Female		Total	
Barriers	**n**	**%**	**n**	**%**	**n**	**%**
**Unaware of cataract**	13	22.4	5	6.6	18	13.4
**Told to wait for cataract to mature**	8	13.8	3	3.9	11	8.2
**Surgical services not available or very**	0	0.0	3	3.9	3	2.2
**Don't know how to get surgery**	0	0.0	2	2.6	2	1.5
**Cannot afford operation**	10	17.2	10	13.2	20	14.9
**No one to accompany**	4	6.9	12	15.8	16	11.9
**No time available/other priorities**	8	13.8	11	14.5	19	14.2
**Old age and need not felt**	2	3.4	3	3.9	5	3.7
**One eye adequate vision/need not felt**	5	8.6	3	3.9	8	6.0
**Fear of operation**	0	0.0	4	5.3	4	3.0
**Fear of loosing eye sight**	0	0.0	5	6.6	5	3.7
**Other disease contra-indicating operation**	7	12.1	12	15.8	19	14.2
**Other**	1	1.7	3	3.9	4	3.0
**Total**	58	100.0	76	100.0	134	100.0

Table 56: Barriers to Cataract Surgery in unilateral NPCB blind with cataract

	Male		Female		Total	
Barriers	n	%	n	%	n	%
**Unaware of cataract**	22	24.7	14	12.2	36	17.6
**Told to wait for cataract to mature**	9	10.1	9	7.8	18	8.8
**Surgical services not available or very**	0	0.0	3	2.6	3	1.5
**Don't know how to get surgery**	2	2.2	3	2.6	5	2.5
**Cannot afford operation**	12	13.5	11	9.6	23	11.3
**No one to accompany**	8	9.0	16	13.9	24	11.8
**No time available/other priorities**	13	14.6	17	14.8	30	14.7
**Old age and need not felt**	5	5.6	3	2.6	8	3.9
**One eye adequate vision/need not felt**	5	5.6	3	2.6	8	3.9
**Fear of operation**	1	1.1	9	7.8	10	4.9
**Fear of loosing eye sight**	0	0.0	7	6.1	7	3.4
**Other disease contra-indicating operation**	10	11.2	17	14.8	27	13.2
**Other**	2	2.2	3	2.6	5	2.5
**Total**	89	100.0	115	100.0	204	100.0

Table 57: Barriers to Cataract Surgery in unilateral cataract with VI (PVA<6/18 in better eye)

	Male		Female		Total	
Barriers	n	%	n	%	n	%
**Unaware of cataract**	25	25.3	16	12.1	41	17.7
**Told to wait for cataract to mature**	10	10.1	12	9.1	22	9.5
**Surgical services not available or very**	0	0.0	4	3.0	4	1.7
**Don't know how to get surgery**	2	2.0	3	2.3	5	2.2
**Cannot afford operation**	11	11.1	11	8.3	22	9.5
**No one to accompany**	8	8.1	17	12.9	25	10.8
**No time available/other priorities**	15	15.2	19	14.4	34	14.7
**Old age and need not felt**	6	6.1	3	2.3	9	3.9
**One eye adequate vision/need not felt**	8	8.1	3	2.3	11	4.8
**Fear of operation**	2	2.0	14	10.6	16	6.9
**Fear of loosing eye sight**	0	0.0	8	6.1	8	3.5
**Other disease contra-indicating operation**	10	10.1	19	14.4	29	12.6
**Other**	2	2.0	3	2.3	5	2.2

**Total**	99	100.0	132	100.0	231	100.0

Table 58: Barriers to Cataract Surgery in people with Bilateral Severe Visual Impairment with cataract

	Male		Female		Total	
Barriers	**n**	**%**	**n**	**%**	**n**	**%**
**Unaware of cataract**	2	50.0	2	33.3	4	40.0
**Don't know how to get surgery**	0	0.0	2	33.3	2	20.0
**No time available/other priorities**	1	25.0	0	0.0	1	10.0
**Old age and need not felt**	1	25.0	0	0.0	1	10.0
**Fear of operation**	0	0.0	1	16.7	1	10.0
**Fear of loosing eye sight**	0	0.0	1	16.7	1	10.0
**Total**	4	100.0	6	100.0	10	100.0

Table 59: Barriers to Cataract Surgery in people with Unilateral Severe Visual Impairment due to cataract

	Male		Female		Total	
Barriers	**n**	**%**	**n**	**%**	**n**	**%**
**Unaware of cataract**	12	26.7	9	17.6	21	21.9
**Told to wait for cataract to mature**	1	2.2	6	11.8	7	7.3
**Surgical services not available or very**	0	0.0	0	0.0	0	0.0
**Don't know how to get surgery**	2	4.4	1	2.0	3	3.1
**Cannot afford operation**	3	6.7	5	9.8	8	8.3
**No one to accompany**	7	15.6	6	11.8	13	13.5
**No time available/other priorities**	5	11.1	8	15.7	13	13.5
**Old age and need not felt**	3	6.7	0	0.0	3	3.1
**One eye adequate vision/need not felt**	2	4.4	0	0.0	2	2.1
**Fear of operation**	1	2.2	8	15.7	9	9.4
**Fear of loosing eye sight**	0	0.0	3	5.9	3	3.1
**Other disease contra-indicating operation**	8	17.8	5	9.8	13	13.5
**Other**	1	2.2	0	0.0	1	1.0
**Total**	45	100.0	51	100.0	96	100.0
